# The BHLHE40‒PPM1F‒AMPK pathway regulates energy metabolism and is associated with the aggressiveness of endometrial cancer

**DOI:** 10.1016/j.jbc.2024.105695

**Published:** 2024-01-30

**Authors:** Kazuo Asanoma, Hiroshi Yagi, Ichiro Onoyama, Lin Cui, Emiko Hori, Minoru Kawakami, Shoji Maenohara, Kazuhisa Hachisuga, Hiroshi Tomonobe, Keisuke Kodama, Masafumi Yasunaga, Tatsuhiro Ohgami, Kaoru Okugawa, Hideaki Yahata, Hiroyuki Kitao, Kiyoko Kato

**Affiliations:** 1 Department of Obstetrics and Gynecology, Faculty of Medical Sciences, Kyushu University, Fukuoka, Japan; 2 Oral Medicine Research Center, Fukuoka Dental College, Fukuoka, Japan

**Keywords:** glycolysis, oxidative phosphorylation, AMPK, BHLHE40, endometrial cancer, phosphoprotein phosphatase

## Abstract

BHLHE40 is a basic helix-loop-helix transcription factor that is involved in multiple cell activities including differentiation, cell cycle, and epithelial-to-mesenchymal transition. While there is growing evidence to support the functions of BHLHE40 in energy metabolism, little is known about the mechanism. In this study, we found that BHLHE40 expression was downregulated in cases of endometrial cancer of higher grade and advanced disease. Knockdown of *BHLHE40* in endometrial cancer cells resulted in suppressed oxygen consumption and enhanced extracellular acidification. Suppressed pyruvate dehydrogenase (PDH) activity and enhanced lactated dehydrogenase (LDH) activity were observed in the knockdown cells. Knockdown of *BHLHE40* also led to dephosphorylation of AMPKα Thr172 and enhanced phosphorylation of pyruvate dehydrogenase E1 subunit alpha 1 (PDHA1) Ser293 and lactate dehydrogenase A (LDHA) Tyr10. These results suggested that BHLHE40 modulates PDH and LDH activity by regulating the phosphorylation status of PDHA1 and LDHA. We found that BHLHE40 enhanced AMPKα phosphorylation by directly suppressing the transcription of an AMPKα-specific phosphatase, PPM1F. Our immunohistochemical study showed that the expression of BHLHE40, PPM1F, and phosphorylated AMPKα correlated with the prognosis of endometrial cancer patients. Because AMPK is a central regulator of energy metabolism in cancer cells, targeting the BHLHE40‒PPM1F‒AMPK axis may represent a strategy to control cancer development.

Endometrial cancer (EC) is the most common gynecological cancer in developed countries including the United States (1). Most EC cases are treated in the early stages and have a favorable prognosis. However, advanced EC cases have limited treatment options and the outcome is extremely poor (2). Thus, there is an urgent need to identify novel diagnostic markers and to develop effective therapeutic strategies for advanced EC.

Basic helix-loop-helix family member e40 (BHLHE40) is a basic helix-loop-helix transcription factor that suppresses the transcription of its target genes by recruiting a histone deacetylase at the class B E-box element of the target genes (3, 4, 5). BHLHE40 is known to be involved in multiple types of cellular activity including apoptosis, senescence, cell cycle, multidrug resistance, and epithelial-to-mesenchymal transition in cancer cells (6, 7, 8, 9, 10). Recently, growing evidence has shown that BHLHE40 regulates the expression of core energy metabolic enzymes and their regulators, including PGC-1a, PPARγ, SREBP-1c, PKLR, FASN, and PCK2 (11, 12, 13, 14, 15, 16). Especially, tissue-resident memory CD8^+^ T cells require Bhlhe40 expression to maintain the tricarboxylic acid (TCA) cycle and oxidative phosphorylation for cell survival (17). However, little is known about energy regulation by BHLHE40 in cancer cells.

AMP-activated protein kinase (AMPK) complex is a serine/threonine kinase that senses energy levels to reprogram cellular metabolism from anabolism to catabolism (18, 19). AMPK deficiency was reported to result in downregulation of pyruvate dehydrogenase (PDH) activity, TCA cycle, oxidative phosphorylation (OXPHOS), fatty acid oxidation, and autophagy, and upregulation of glycolysis, extracellular acidification rate, lactate production, and LDH activity (20, 21, 22, 23, 24). AMPK also plays a critical role in cancer development by regulating cell proliferation, epithelial-to-mesenchymal transition, multidrug resistance, and stemness gene expression as well as energy homeostasis (24, 25, 26, 27, 28).

The metal-dependent protein phosphatase (PPM) family is composed of 20 serine/threonine phosphatase isoforms. PPMs bind to manganese/magnesium ions (Mn^2+^/Mg^2+^) in their catalytic cores and act as single-subunit enzymes. Substrate-specific phosphatase function was reported for each isoform (29). Especially, PPM1A, PPM1B, PPM1E, and PPM1F are reported to specifically dephosphorylate AMPKα Thr172 (30, 31, 32, 33).

In this study, we investigated the novel BHLHE40–PPM1F–AMPK pathway in the regulation of energy metabolism in the development of EC cells.

## Results

### BHLHE40 regulated the phosphorylation of AMPKα

**Figure 1:**
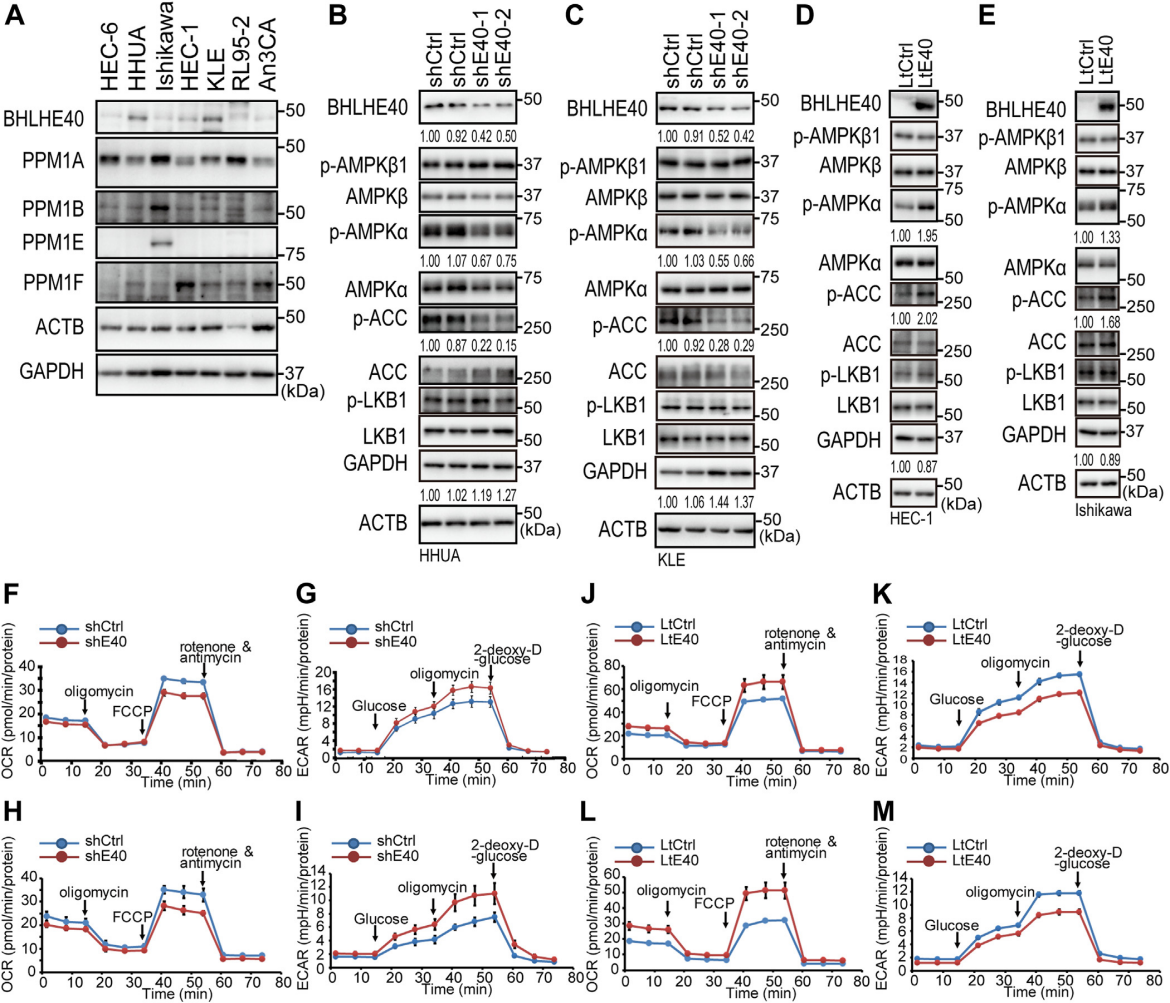
**BHLHE40 affected phosphorylation of AMPKα at Ser172, OCRs, and ECARs.***A*, gene expression profiles of BHLHE40, PPM1A, PPM1B, PPM1E, and PPM1F in EC cell lines by immunoblotting. ACTB and GAPDH were used as internal controls. *B–E*, BHLHE40 was knocked down in HHUA (*B*) and KLE (*C*) cells using two different shRNA constructs (9). BHLHE40 was overexpressed in HEC-1 (*D*) and Ishikawa (*E*) cells. *B–E*, values under panels indicate the relative expression levels of BHLHE40/ACTB, GAPDH/ACTB, p-AMPKα/AMPKα, and p-ACC/ACC. *A*, Data are representative of two technical replicates. *B–E*, Data are representative of at least three biological replicates. Extracellular flux analysis of HHUA (*F* and *G*), KLE (*H* and *I*), HEC-1 (*J* and *K*), and Ishikawa (*L* and *M*) cells. Real-time OCRs (*F*, *H*, *J*, and *L*) and ECARs (*G*, *I*, *K*, and *M*) were measured upon treatment with the indicated inhibitors or glucose. *F–M*, data are from three technical replicates. The experiments were biologically replicated twice and representative data are shown. LtCtrl, control lentiviral vector; LtE40, lentiviral vector to express BHLHE40; shCtrl, control shRNA; shE40, shRNA to knockdown BHLHE40 expression.

To study the impact of BHLHE40 expression in the energy metabolism of EC, we first assessed whether BHLHE40 regulated the activity of AMPK, a central regulator of energy metabolism. BHLHE40 is expressed in HHUA and KLE EC cells and is absent in the other cell lines examined (Fig. 1*A*). While the expression and phosphorylation of AMPKβ1 was not affected following the knockdown of *BHLHE40* in HHUA and KLE cells, AMPKα phosphorylation at Thr172 (p-AMPKα) was downregulated (Fig. 1, *B* and *C*). The phosphorylation of an AMPK target enzyme, ACC Ser79, was also remarkably suppressed in the knockdown cells (Fig. 1, *B* and *C*). However, forced expression of BHLHE40 enhanced the phosphorylation of AMPKα and ACC (Fig. 1, *D* and *E*). LKB1 is a well-known direct kinase of AMPKα phosphorylation at Thr172. Because direct negative regulation of LKB1 by BHLHE40 was reported elsewhere, we examined the expression of LKB1 (34). However, total LKB1 and phosphorylated LKB1 Ser428 were not altered by modulation of BHLHE40 expression (Fig. 1, *B*–*E*). Interestingly, the protein levels of GAPDH were enhanced by knockdown of *BHLHE40* and suppressed by forced expression of BHLHE40 (Fig. 1, *B*–*E*).

Because AMPK has been reported to regulate glycolysis and OXPHOS (21, 22, 23), we applied the EC cells to a flux analyzer. Knockdown of *BHLHE40* resulted in downregulation of the oxygen consumption rate (OCR) (Fig. 1, *F* and *H*) and upregulation of the extracellular acidification rate (ECAR) (Fig. 1, *G* and *I*). Conversely, forced expression of BHLHE40 resulted in upregulation of the OCR (Fig. 1, *J* and *L*) and downregulation of the ECAR (Fig. 1, *K* and *M*). These series of results suggested that BHLHE40 activated AMPK followed by upregulation of OXPHOS.

### Comprehensive analysis of the downstream pathway of BHLHE40

**Figure 2:**
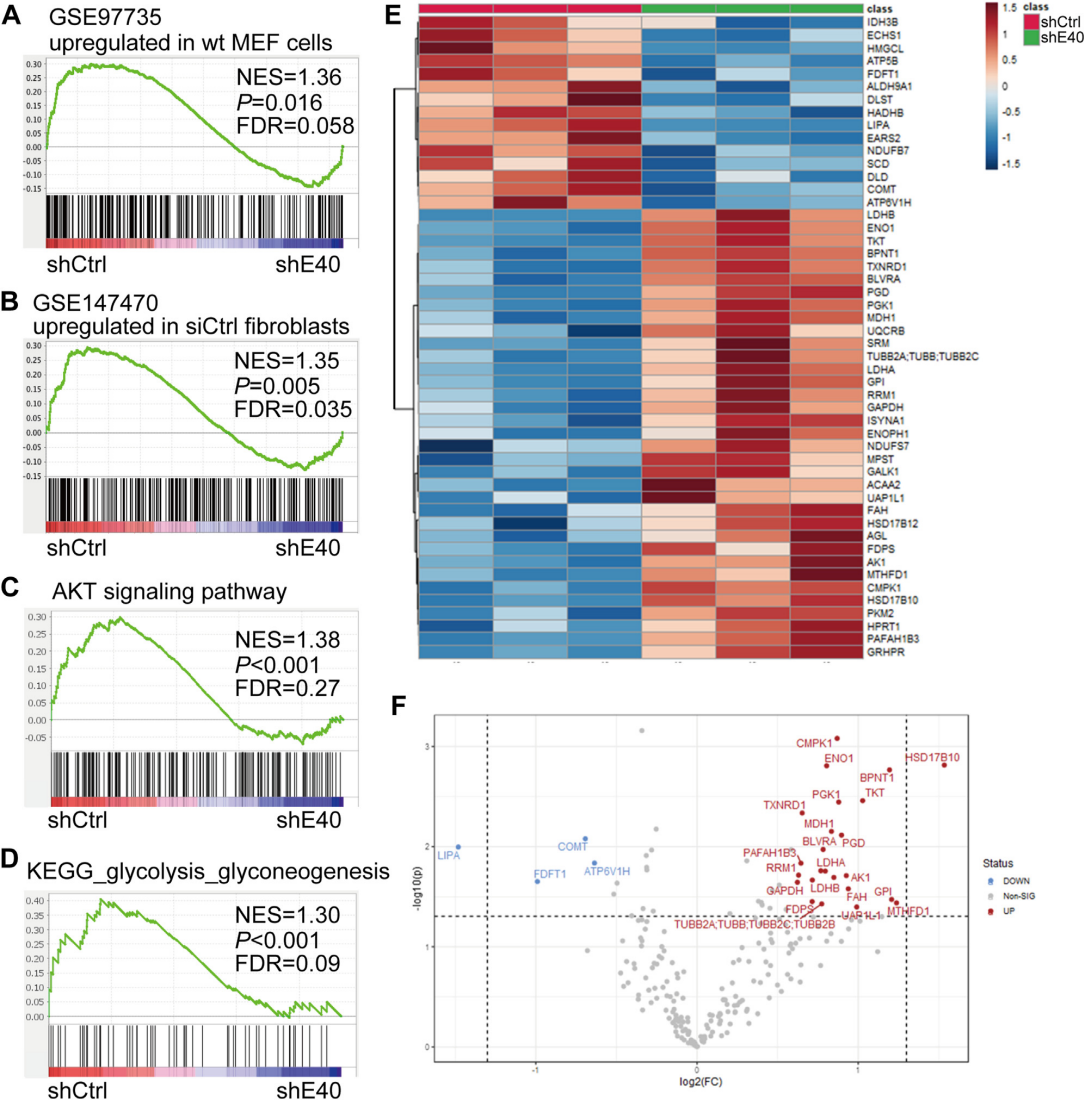
**Comprehensive analysis of BHLHE40-knocked down HHUA cells by microarray and iMPAQT analyses.***A*, Gene Set Enrichment Analysis (GSEA) of upregulated genes in wild-type mouse embryonic fibroblasts (MEFs) and *AMPKα1* and *AMPKα2* double knockout MEFs (https://www.ncbi.nlm.nih.gov/geo/: accession number GSE97735) were compared with upregulated genes in shCtrl-transfected HHUA cells relative to those transfected with shBHLHE40. *B*, GSEA of upregulated genes in control cardiac fibroblasts transfected with scrambled siRNA and those transfected with siAMPKα1 (https://www.ncbi.nlm.nih.gov/geo/: accession number GSE147470) were compared with upregulated genes in HHUA cells transfected with shCtrl relative to those transfected with shBHLHE40. *C*, an annotated gene set of 187 genes upregulated in AKT-transgenic murine prostate (M2666, MSigDB, https://www.gsea-msigdb.org/gsea/msigdb/) was compared with upregulated genes in HHUA cells transfected with shCtrl relative to those transfected with shBHLHE40. *D*, an annotated gene set of 62 genes from the glycolysis/gluconeogenesis pathway by Kyoto Encyclopedia of Genes and Genomes (KEGG) analysis (M11521, MSigDB, https://www.gsea-msigdb.org/gsea/msigdb/) was compared with upregulated genes in HHUA cells transfected with shCtrl relative to those transfected with shBHLHE40. *E*, a clustered heat map analysis of iMPAQT data. *F*, Volcano plotting analysis of iMPAQT data. Both microarray and iMPAQT data were from three biological replicates. shCtrl, shControl; shE40, shBHLHE40.

As shown above, because BHLHE40 was suggested to regulate energy metabolism mediated by AMPK activity, we applied the mRNA and protein from control and *BHLHE40*–knockdown HHUA cells to microarray and proteome analyses, respectively. As expected, gene set enrichment analysis (GSEA) of the microarray showed that the control HHUA cells exhibited enrichment of the AMPK pathway gene signature compared with *BHLHE40*–knockdown cells (Fig. 2, *A* and *B*). Furthermore, the control cells also exhibited gene enrichment of the AKT pathway and glycolysis–glyconeogenesis pathway compared with the knockdown cells (Fig. 2, *C* and *D*). Absolute quantitative proteomic analysis by iMPAQT (*in vitro* proteome-assisted MRM for Protein Absolute QuanTification) was used for the investigation of metabolism pathways by BHLHE40 (35). As expected, the knockdown of *BHLHE40* enhanced the expression of enzymes such as GPI, PGK-1, LDH, GAPDH, ENO, PGD, and TKT, involving the glycolysis and pentose phosphate pathways (Fig. 2, *E* and *F*).

### BHLHE40 regulated PDH and LDH activity by modulating phosphorylation of PDHA1 and LDHA

**Figure 3:**
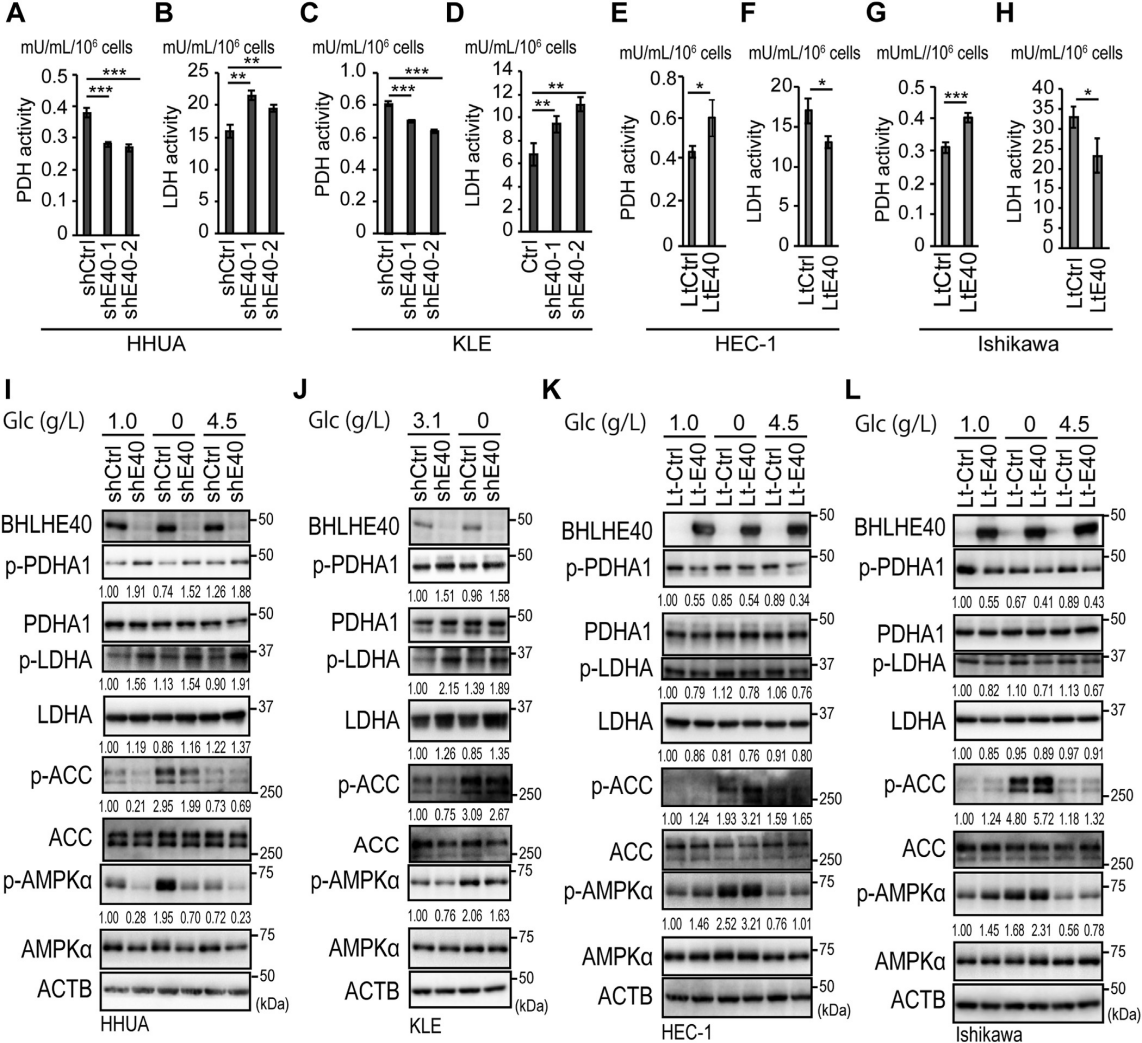
**PDH and LDH activity and PDHA1 and LDHA expression in BHLHE40-modulated EC cells.** PDH activity (*A*, *C*, *E*, and *G*) and LDH activity (*B*, *D*, *F*, and *H*) were measured in HHUA (*A* and *B*), KLE (*C* and *D*), HEC-1 (*E* and *F*), and Ishikawa (*G* and *H*) cells after culturing for 24 h in DMEM or DMEM:F12 with 10% FBS with 1 mM sodium pyruvate without glucose. *A–H*, data are from three technical replicates. The experiments were biologically replicated three times and representative data are shown. *I–L*, immunoblotting analysis of EC cells cultured for 24 h in DMEM or DMEM:F12 with the indicated concentrations of glucose with 1 mM sodium pyruvate and 10% FBS. HHUA (*I*), KLE (*J*), HEC-1 (*K*), and Ishikawa (*L*) cells. *I–L*, values under panels indicate the relative expression levels of p-PDHA1/PDHA1, p-LDHA/LDHA, p-AMPKα/AMPKα, p-ACC/ACC, and LDHA/ACTB. Data are representative of at least three biological replicates. shCtrl, shControl; shE40, shBHLHE40; LtCtrl, LtControl; LtE40, LtBHLHE40. *A–H*, unpaired two-sided Student’s *t* test or the Mann–Whitney *U* test was used. ∗*p* < 0.05; ∗∗*p* < 0.01; ∗∗∗*p* < 0.001.

The above results suggested that BHLHE40 regulates glycolysis and OXPHOS by modulating AMPK activity. Knockdown of *BHLHE40* suppressed p-AMPKα and forced expression of BHLHE40 enhanced it regardless of glucose concentration (Fig. 3, *I*–*L*). AMPK has been reported to suppress the phosphorylation of PDHA1 (Ser293) and upregulate PDH activity (20, 36). However, AMPK suppressed LDH activity and lactate production (21, 24). We first examined PDH and LDH activity in *BHLHE40*–knockdown cells (Fig. 3, *A*–*D*) and BHLHE40-overexpressing cells (Fig. 3, *E*–*H*). As expected, while knockdown of *BHLHE40* suppressed PDH activity and enhanced LDH activity (Fig. 3, *A*–*D*), forced expression of BHLHE40 enhanced PDH activity and suppressed LDH activity (Fig. 3, *E*–*H*). Parallel to LDH activity, lactate production was enhanced by *BHLHE40*–knockdown and suppressed by BHLHE40 overexpression (Fig. S1). Inhibitory phosphorylation of Ser293 of PDHA1 was enhanced by *BHLHE40* knockdown and suppressed by BHLHE40 overexpression (Fig. 3, *I*–*L*). Furthermore, phosphorylation of Tyr10 of LDHA was also enhanced by *BHLHE40* knockdown and suppressed by BHLHE40 overexpression (Fig. 3, *I*–*L*). Consistent with the iMPAQT data, protein levels of LDHA were enhanced by *BHLHE40* knockdown and suppressed by forced expression of BHLHE40 (Fig. 3, *I*–*L*).

### BHLHE40 affected glycolysis, OXPHOS, PDH, and LDH activity mediated by regulation of AMPKα

**Figure 4:**
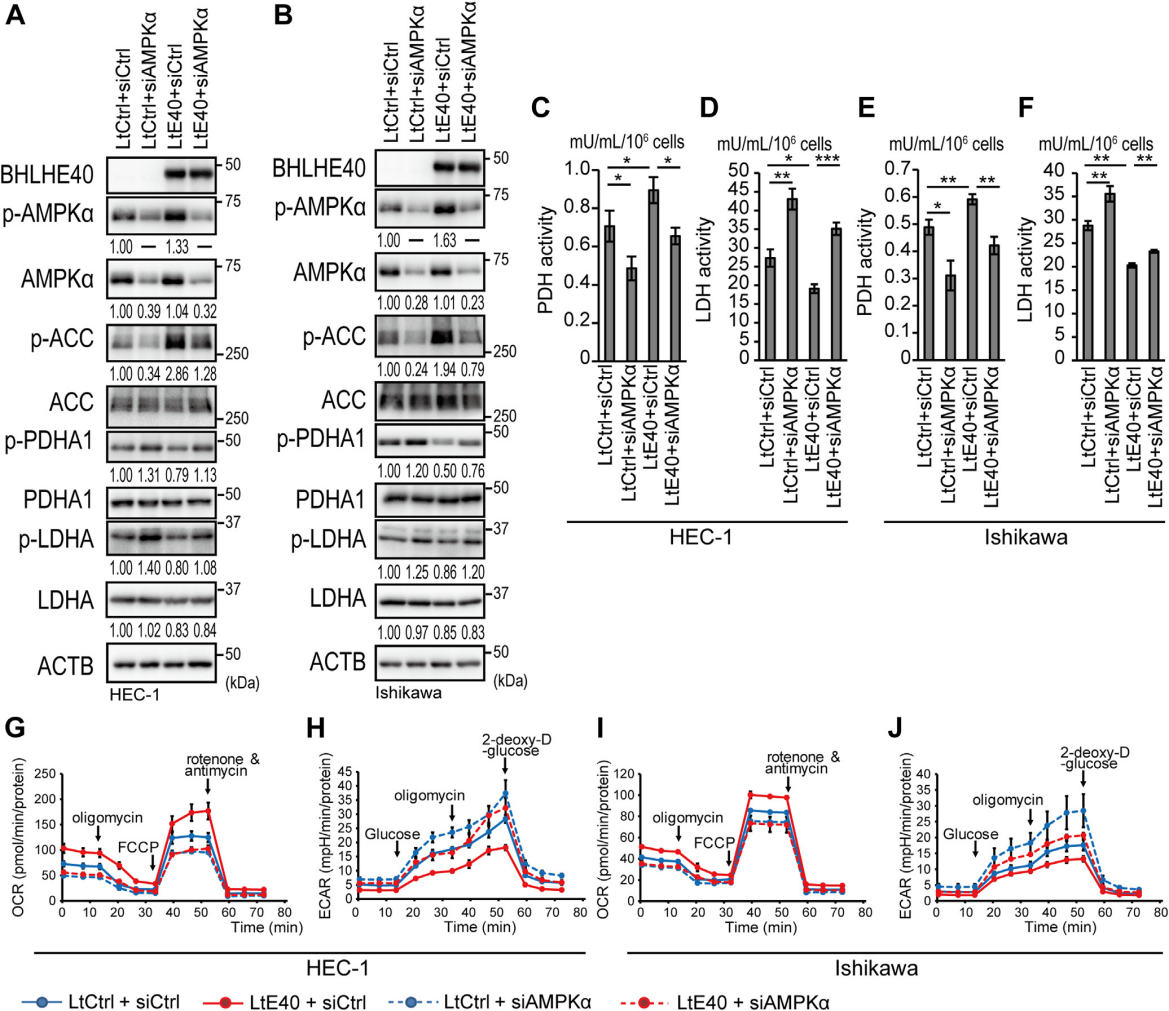
**Impact of AMPKα expression on BHLHE40-expressing EC cells.** Immunoblotting analysis of EC cells transfected with an siRNA against *AMPKα1/2* (siAMPKα) and cultured for 24 h in DMEM with 10% FBS and 1 mM sodium pyruvate without glucose. HEC1 (*A*) and Ishikawa (*B*) cells. *A* and *B*, values under panels indicate relative expression levels of p- AMPKα/AMPKα, p-ACC/ACC, p-PDHA1/PDHA1, p-LDHA/LDHA, AMPKα/ACTB and LDHA/ACTB. *A* and *B*, data are representative of at least three biological replicates. PDH activity (*C* and *E*) and LDH activity (*D* and *F*) were measured in HEC1 (*C* and *D*) and Ishikawa (*E* and *F*) cells after culturing for 24 h in DMEM with 10% FBS and 1 mM sodium pyruvate without glucose. (*C*–*F*) Data are from three technical replicates. The experiments were biologically replicated three times and representative data are shown. Extracellular flux analysis of HEC-1 (*G* and *H*) and Ishikawa (*I* and *J*) cells. Real-time OCRs (*G* and *I*) and ECARs (*H* and *J*) were measured upon treatment with the indicated inhibitors or glucose. *G–J*, data are from three technical replicates. The experiments were biologically replicated twice and representative data are shown. *C–F*, unpaired two-sided Student’s *t* test or the Mann–Whitney *U* test was used. ∗*p* < 0.05; ∗∗*p* < 0.01; ∗∗∗*p* < 0.001. LtCtrl, LtControl; LtE40, LtBHLHE40; shCtrl, shControl; shE40, shBHLHE40.

The results above suggested that AMPKα regulated by BHLHE40 affected glycolysis, OXPHOS, PDH, and LDH activity. To verify this hypothesis, we knocked down *AMPKα* in Ishikawa and HEC-1 cells, in which p-AMPKα was upregulated by forced expression of BHLHE40 (Fig. 4, *A* and *B*). Knockdown of *AMPKα* enhanced phosphorylation of Ser293 of PDHA1 and Tyr10 of LDHA (Fig. 4, *A* and *B*). Consistent with the iMPAQT data, protein levels of LDHA were enhanced by BHLHE40 expression but were not altered by *AMPKα* knockdown (Fig. 4, *A* and *B*). Furthermore, suppressed phosphorylation of PDHA1 and LDHA by BHLHE40 expression was attenuated by knockdown of *AMPKα*. As expected, knockdown of *AMPKα* suppressed PDH activity and enhanced LDH activity (Fig. 4, *C*–*F*). Furthermore, enhanced PDH activity and suppressed LDH activity by forced expression of BHLHE40 were attenuated by knockdown of *AMPKα* (Fig. 4, *C*–*F*). Flux analyzer analysis showed that knockdown of *AMPKα* suppressed OCR and enhanced ECAR (Fig. 4, *G*–*J*). Similarly, enhanced OCR and suppressed ECAR resulting from forced expression of BHLHE40 were attenuated by knockdown of *AMPKα* (Fig. 4, *G*–*J*).

### BHLHE40 suppressed the expression of PPM1A and PPM1F

**Figure 5:**
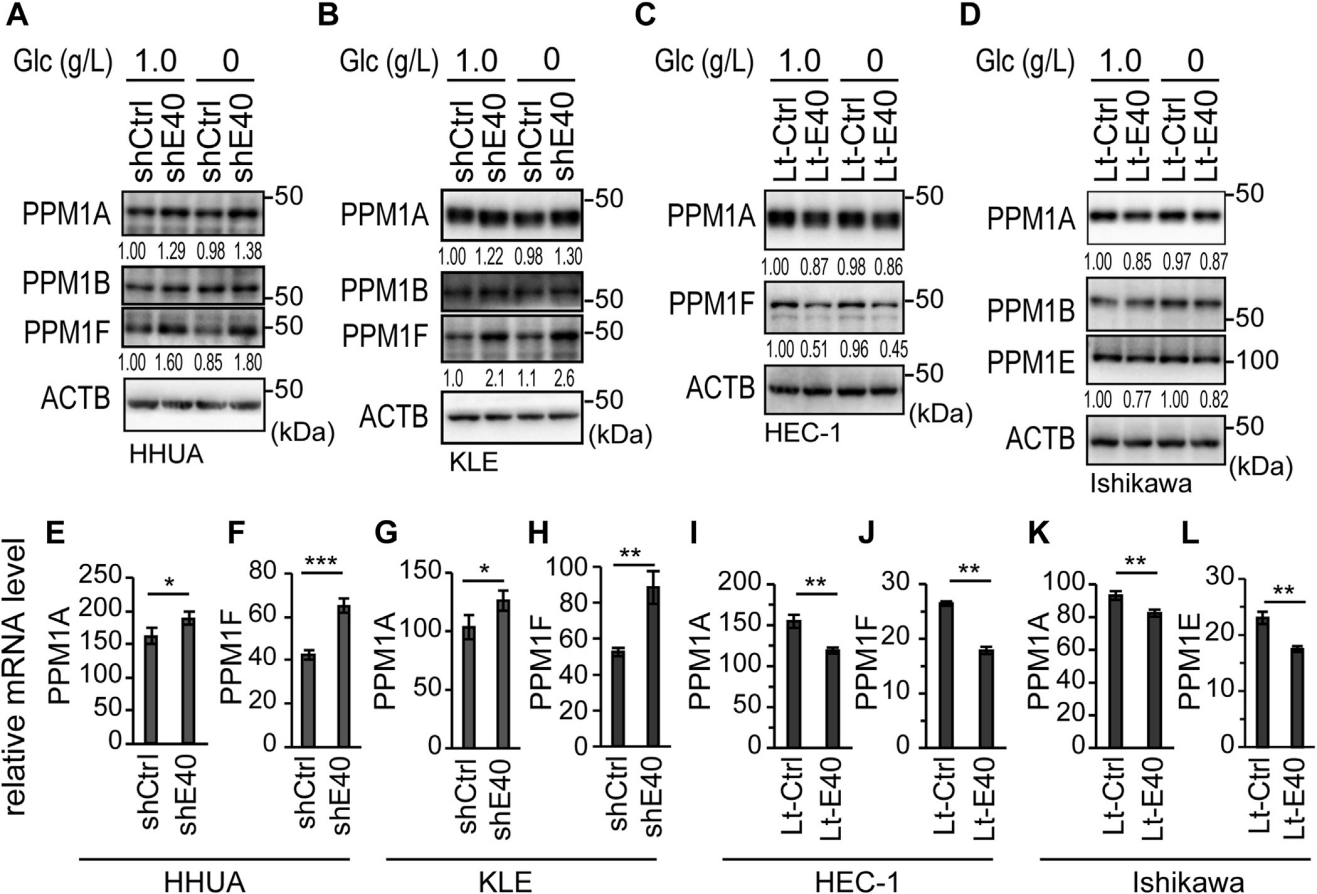
**Expression of PPM1s in BHLHE40-modulated EC cells.***A–D*, immunoblotting analysis of EC cells cultured for 24 h in DMEM or DMEM:F12 with the indicated concentrations of glucose with 1 mM sodium pyruvate and 10% FBS. HHUA (*A*), KLE (*B*). HEC-1 (*C*), and Ishikawa (*D*) cells. *A–D*, values under panels indicate relative expression levels of PPM1A/ACTB, PPM1F/ACTB, and PPM1E/ACTB. *A–D*, data are representative of three biological replicates. *E–L*, real-time RT-PCR analysis of EC cells cultured for 24 h in DMEM or DMEM:F12 with 1.0 g/L glucose, 1 mM sodium pyruvate, and 10% FBS. HHUA (*E* and *F*), KLE (*G* and *H*). HEC-1 (*I* and *J*), and Ishikawa (*K* and *L*) cells. PPM1A (*E*, *G*, *I*, and *K*), PPM1F (*F*, *H*, and *J*), and PPM1E (*L*). *E–L*, data are from three biological replicates. Unpaired two-sided Student’s *t* test was used. ∗*p* < 0.05; ∗∗*p* < 0.01; ∗∗∗*p* < 0.001. LtCtrl, LtControl; LtE40, LtBHLHE40; shCtrl, shControl; shE40, shBHLHE40.

Next, we focused on the mechanism of positive regulation of AMPKα phosphorylation by BHLHE40. To examine the possibility that BHLHE40 affected the [AMP + ADP]/ATP ratio to regulate the phosphorylation of AMPKα Ser172, we assayed the ADP/ATP ratio in *BHLHE40*-knockdown or BHLHE40-overexpressing EC cells. There were no significant differences in ADP/ATP ratio in the cells in which BHLHE40 was modulated (Fig. S2). The metal-dependent protein phosphatases PPM1A, PPM1B, PPM1E, and PPM1F are reported to dephosphorylate AMPKα (30, 31, 32, 33). Various patterns of PPM1A, PPM1B, PPM1E, and PPM1F expression were observed in EC cell lines (Fig. 1*A*). To confirm whether BHLHE40 regulates the expression of PPM1s, *BHLHE40*-knockdown or BHLHE40-overexpressing EC cells were examined for their expression of PPM1s. Knockdown of *BHLHE40* in HHUA (Fig. 5, *A*, *E*, and *F*) and KLE (Fig. 5, *B*, *G*, and *H*) cells resulted in upregulation of PPM1A and PPM1F protein (Fig. 5, *A* and *B*) and mRNA (Figs. S3, *A*–*D* and 5, *E*–*H*). In both cell lines, the upregulation of PPM1F was more remarkable than that of PPM1A (Fig. 5, *A*, *B*, and *E*–*H*). Conversely, forced expression of BHLHE40 in HEC-1 (Fig. 5, *C*, *I*, and J) and Ishikawa (Fig. 5, *D*, *K*, and *L*) cells resulted in downregulation of PPM1A, PPM1E, and PPM1F protein (Fig. 5, *C* and *D*) and mRNA (Fig. 5, *I*–*L*). Similar to the knockdown, PPM1F expression changed more remarkably than that of PPM1A or PPM1E (Fig. 5, *C*, *D*, *I*, and *J*).

### BHLHE40 suppressed the expression of PPM1A and PPM1F by transcriptional regulation

**Figure 6:**
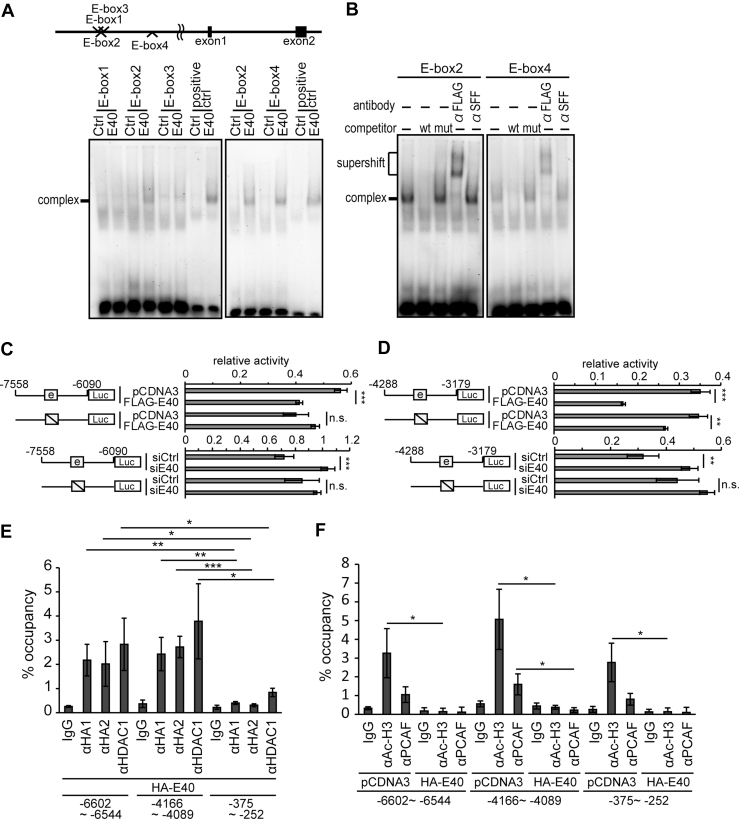
**BHLHE40 transcriptionally suppresses PPM1F expression.***A, top*, schematic presentation of four E-boxes in the promoter of *PPM1F*. *A, bottom*, Gel shift assay using nuclear extracts from 293T cells transfected with FLAG-BHLHE40 was incubated with labeled E-box1, 2, 3, and 4 probes (Table S4). A canonical E-box probe from the *BHLHE41* promoter was used as a positive control (58). *B*, anti-FLAG antibody was used to form supershifted bands. An anti-SRF antibody was used as a negative control. *A* and *B*, data are representative of two biological replicates. *C* and *D, top*, reporter analysis of the wild type and mutant *PPM1F* promoter in HEC-6 cells transfected with FLAG-BHLHE40 (Table S3). *C* and *D, bottom*, Reporter analysis of the wild type and mutant *PPM1F* promoter in HHUA cells transfected with siBHLHE40 at a concentration of 50 nM. See also Fig. S6. *C* and *D*, data are from four technical replicates. The experiments were biologically replicated three times and representative data are shown. *E* and *F*, ChIP assay using 293T cells transfected with empty vector (pCDNA3) or HA-BHLHE40 (pCDNA3-HA-BHLHE40). Protein–DNA complexes immunoprecipitated with anti-HA, anti-HDAC1, anti-acetylated-histone H3 (Ac-H3), or anti-PCAF antibodies were used to amplify indicated promoter regions by PCR (Table S1). The −6602 to −6544 and −4166 to −4089 regions contain E-Box2 and E-Box4, respectively. The −375 to −252 region represents a negative control. The occupancy ratios (%) were calculated using 10% input samples as standards. αHA1, anti-HA (HA-7; Sigma–Aldrich) antibody; αHA2, anti-HA (ab9110; Abcam) antibody. *E* and *F*, data are from three biological replicates. (*C–F*) Unpaired two-sided Student’s *t* test or the Mann–Whitney *U* test was used. ∗*p* < 0.05; ∗∗*p* < 0.01; ∗∗∗*p* < 0.001.

To explore the suppressive mechanism of PPM1s by BHLHE40, we focused on transcriptional regulation. By searching the upstream promoter region of PPM1s, several perfect canonical E-boxes (-CACGTG-) were found in PPM1A and PPM1F, but not in PPM1B and PPM1E (Figs. S4*A* and 6*A*, upper schemas). To examine the affinity of BHLHE40 to the canonical E-box, nuclear extracts from 293T cells expressing FLAG-labelled BHLHE40 were used to form DNA–protein complexes. Among two E-boxes (E-box1 and E-box2) in the promoter of *PPM1A*, the E-box1 was found to bind to BHLHE40 (Fig. S4*A*, lower panel). On the other side, among four E-boxes (E-box1∼4) in the promoter of *PPM1F*, the E-box2 and 4 bound to BHLHE40 (Fig. 6*A*, lower panels). The specific binding of BHLHE40 was confirmed by supershift formation by adding anti-FLAG antibody (Figs. S4*B* and 6*B*). A reporter assay was performed using the upstream promoter region (−1098–+820 bp) of *PPM1A*. As expected, the reporter activity was suppressed by forced expression of BHLHE40 (Fig. S4*C*, upper graph). However, the knockdown of *BHLHE40* enhanced the reporter activity (Fig. S4*C*, lower graph). The introduction of a mutation in the E-box1 diminished the effects (Fig. S4*C*). Two separate fragments of the *PPM1F* promoter regions (−7558 to -6090 bp and −4288 to −3179 bp) possessing the E-box2 and E-box4, respectively, were used for a reporter assay. Again, as expected, the reporter activity was remarkably suppressed by the forced expression of BHLHE40 (Fig. 6, *C* and *D*, upper graphs). Conversely, the knockdown of *BHLHE40* enhanced the reporter activity (Fig. 6, *C* and *D*, lower graphs). The introduction of mutations in the E-box2 and E-box4, respectively, diminished these effects (Fig. 6, *C* and *D*).

Next, we applied a chromatin immunoprecipitation (ChIP) assay to examine the association between BHLHE40 and the promoter regions of *PPM1A* and *PPM1F*. For ChIP assay, we first used HHUA cells which endogenously express BHLHE40. We tested three anti-BHLHE40 antibodies (sc-101023 from Santa Cruz Biotechnology; HPA028921 from Atlas Antibodies; NB100 to 1800 from Novus Biologicals) in the ChIP assay. However, none of these antibodies worked in our systems. Then we used 293T cells overexpressing HA-BHLHE40 in the ChIP assay to demonstrate the interaction of BHLHE40 and the DNA promoter regions of *PPM1A* and *PPM1F* containing canonical E-boxes (Figs. S4*D* and 6*E*). While the *PPM1F* promoter regions containing E-box2 (−6602 to −6544 bp) and E-box4 (−4166 to −4089 bp) were specifically associated with BHLHE40, the proximal region (−375 to −252 bp) was not (Fig. 6*E*). The binding of BHLHE40 to the promoter regions accompanied HDAC1 binding (Fig. 6*E*) (37, 38, 39). Furthermore, compared with the control cells, forced expression of BHLHE40 excluded acetylated histone H3 and PCAF from the promoter regions (Fig. 6*F*). In contrast, the *PPM1A* promoter region showed no specific binding to BHLHE40 and HDAC1 regardless of the involvement of E-box1 (−1006 to −870 bp) (Fig. S4*D*). Specific binding of acetylated histone H3 and PCAF was not observed in the absence of BHLHE40 expression (Fig. S4*E*).

### PPM1A and PPM1F specifically dephosphorylated AMPKα

**Figure 7:**
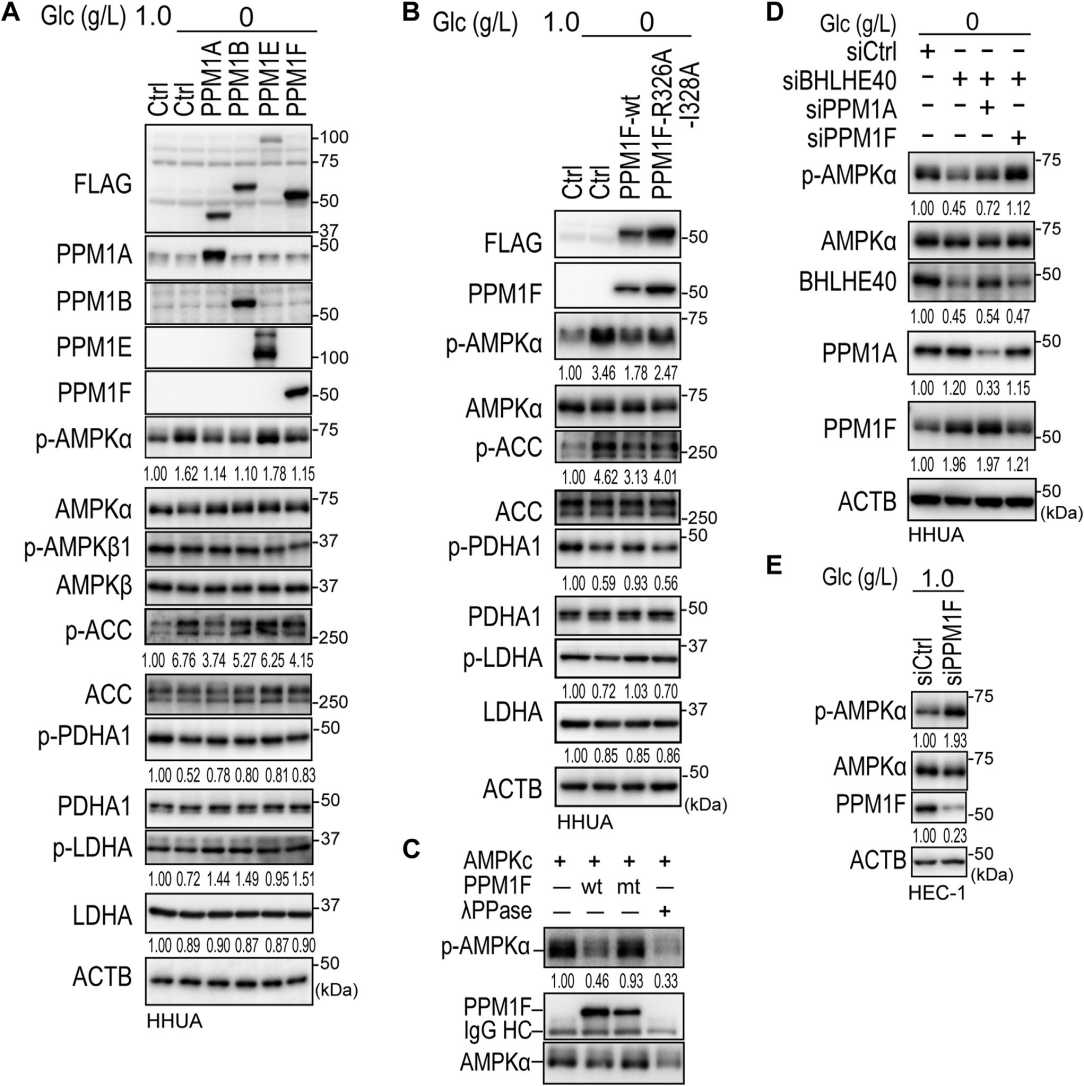
**Phosphatase activity of PPM1F on phospho-AMPKα Ser172.***A*, immunoblotting of HHUA cells transfected with FLAG-tagged PPM1A-, PPM1B-, PPM1E-, or PPM1F-expressing vector. *B*, immunoblotting of HHUA cells transfected with wild type and phosphatase inactive mutant (R326A-I328A) PPM1F. *C*, *in vitro* phosphatase assay reconstituted with FLAG-PPM1F and activated AMPK complex. *D* and *E*, immunoblotting analysis of HHUA (*D*) and HEC-1 (*E*) cells transfected with indicated siRNAs. Values under panels indicate relative expression levels of p-AMPKα/AMPKα, p-ACC/ACC, p-PDHA1/PDHA1, p-LDHA/LDHA, LDHA/ACTB, BHLHE40/ACTB, PPM1A/ACTB, and PPM1F/ACTB. Data are representative of at least two technical replicates from three biological replicates.

Because PPM1A, PPM1B, PPM1E, and PPM1F were reported to dephosphorylate AMPKα (30, 31, 32, 33), HHUA cells, which showed only low expression of all four PPM1s, were used to overexpress each of the four phosphatases (Fig. 1*A*). All the PPM1s except PPM1E dephosphorylated AMPKα at Thr172 to some extent (Fig. 7*A*). Interestingly, phosphorylation of PDHA1 at Ser293 was enhanced by PPM1s expression (Fig. 7*A*). Similarly, phosphorylation of LDHA at Tyr10 was also enhanced by PPM1s expression (Fig. 7*A*). To investigate the specific phosphatase activity of PPM1A and PPM1F against AMPKα, phosphatase dead mutants of PPM1A and PPM1F were constructed. R174G mutation and R326A/I328R mutation were reported to inactivate the phosphatase activity of PPM1A and PPM1F, respectively (40, 41). While the wild type of PPM1A and PPM1F dephosphorylated AMPKα Thr172 and its direct target ACC at Ser79, R174G mutant of PPM1A and R326A/I328R mutant of PPM1F failed to dephosphorylate them (Figs. S5*A* and 7*B*). Furthermore, while phosphorylation of PDHA1 Ser293 and LDHA Tyr10 was enhanced by wild type PPM1A and PPM1F, the effect was alleviated by the mutant types (Figs. S5*A* and 7*B*). An *in vitro* phosphatase assay also showed that wild-type PPM1A and PPM1F dephosphorylated AMPKα, while the mutant types failed to dephosphorylate it (Figs. S5*B* and 7*C*). These results suggested that PPM1A and PPM1F directly dephosphorylated AMPKα.

### PPM1F regulated the phosphorylation of AMPKα downstream of BHLHE40

To confirm that PPM1A and PPM1F regulated the phosphorylation of AMPKα downstream of BHLHE40, combined knockdown of *BHLHE40* and *PPM1A* or *BHLHE40* and *PPM1F* was performed in HHUA cells. Knockdown of *BHLHE40* resulted in dephosphorylation of AMPKα accompanied by upregulation of PPM1F (Fig. 7*D*). Impressively, the upregulation of PPM1A was less than that of PPM1F, which was consistent with Figure 5 (Fig. 7*D*). Furthermore, the knockdown of *PPM1F* had a larger effect on the phosphorylation of AMPKα than the knockdown of *PPM1A* (Fig. 7*D*).

### Knockdown of PPM1s enhanced the phosphorylation of AMPKα

Various patterns of PPM1A, PPM1B, PPM1E, and PPM1F expression were observed in EC cell lines (Fig. 1*A*). HEC-6 and HEC-1 cells exhibited dominant expression of PPM1A and PPM1F, respectively (Fig. 1*A*). Interestingly, Ishikawa cells expressed PPM1A, PPM1B, and PPM1E at the same time (Fig. 1*A*). Knockdown of *PPM1A* in HEC-6 and *PPM1F* in HEC-1 resulted in prominent phosphorylation of AMPKα at Thr172 (Figs. S5*C* and 7*E*). Furthermore, knockdown of *PPM1A* and *PPM1B* in Ishikawa cells resulted in phosphorylation of AMPKα, but knockdown of *PPM1E* had no significant effect (Fig. S5*D*). This result was consistent with that in Figure 7*A* showing that forced expression of PPM1E had no significant effect on AMPKα phosphorylation.

### Protein expression levels of BHLHE40, p-AMPKα, and PPM1F correlated with each other in EC tissue samples

**Figure 8:**
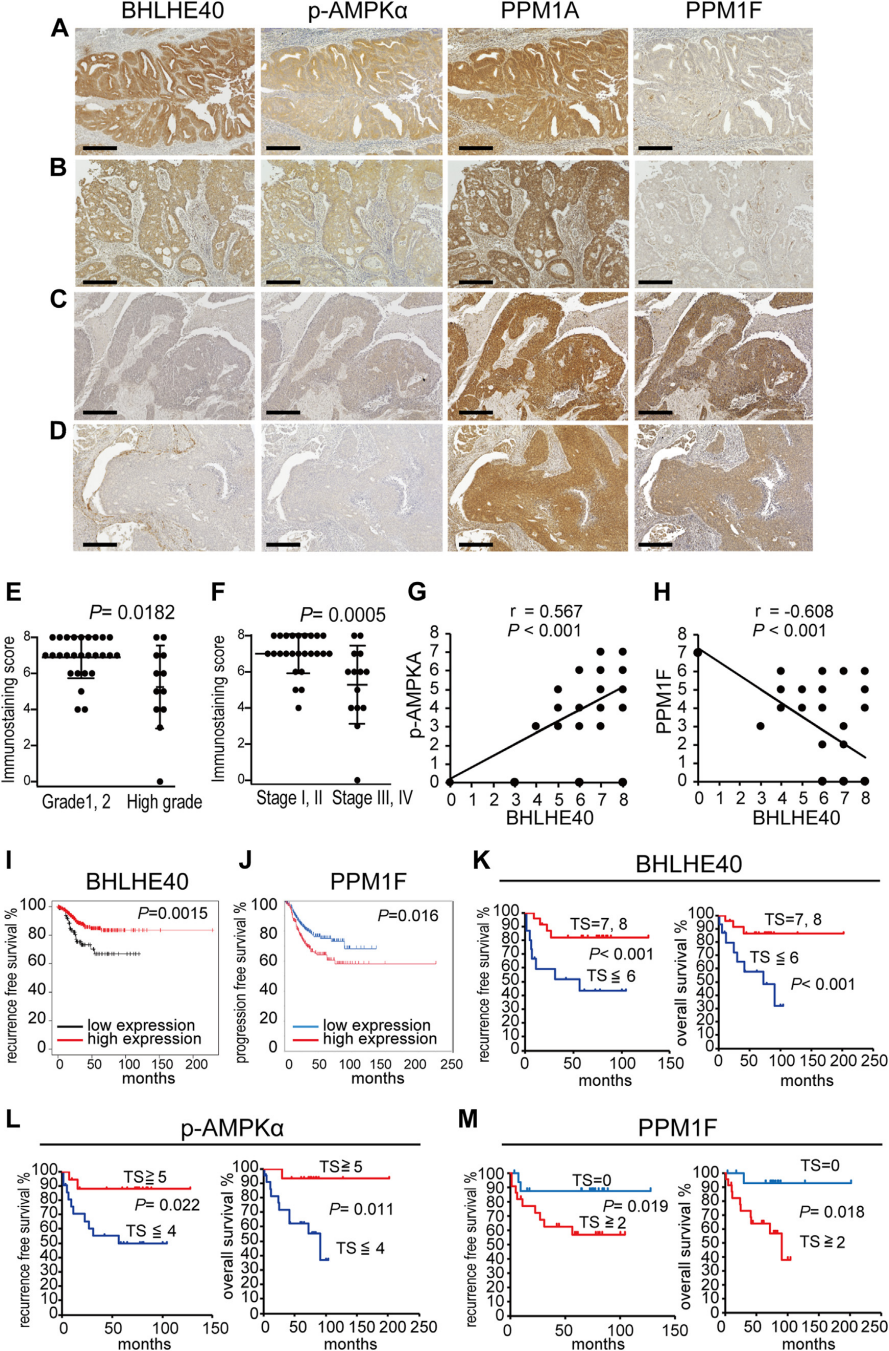
**Protein expression of BHLHE40, phospho-AMPKα, and PPM1F correlates with prognosis of endometrial cancer patients.***A–D*, Immunohistochemical analysis of primary sites from surgically removed uteri. Stage IA, endometrioid carcinoma, grade 1 (*A*); stage IB, endometrioid carcinoma grade 1 (*B*); stage IVB, endometrioid carcinoma, grade 3 (*C*); stage IIIC2, serous carcinoma (*D*). The scale bars indicate 200 μm. Total staining score of BHLHE40 was evaluated between groups of endometrioid carcinoma grade 1 and 2 and that of grade 3, serous carcinoma (*E*). Comparison of groups at stage I and II and that of stage III and IV (*F*). *E* and *F*, Welch’s *t* test was applied. *G*, total staining scores of BHLHE40 and phospho-AMPKα were analyzed using Pearson’s product-moment correlation coefficient. Also see Fig. S7*A*. *H*, Total staining scores of BHLHE40 and PPM1F were analyzed using Pearson’s product-moment correlation coefficient. Also see Fig. S7*B*. r-values show correlation coefficients. *I*, correlation between *BHLHE40* mRNA levels and recurrence-free survival of endometrial cancer cases (n = 543 from Gene Expression Omnibus, European Genome-Phenome Archive, and TCGA databases) was analyzed by KM potter (http://kmplotter.com/analysis/). *J*, correlation between *PPM1F* mRNA and progression-free survival from TCGA (n = 505) was visualized by the Kaplan–Meier curve and evaluated by the log-rank test. Correlation between total staining score of BHLHE40 (*K*), phospho-AMPKα (*L*), PPM1F (*M*), and prognosis (*left panels*, recurrence-free survival; *right panels*, overall survival) of patients (*n* = 39) was visualized by a Kaplan–Meier curve and evaluated by the log-rank test.

Protein expression was analyzed using primary tissue samples of EC by IHC analysis (Fig. 8, *A*–*D*). Thirty-nine primary EC tissue samples were analyzed for BHLHE40, p-AMPKα, PPM1A, and PPM1F. As reported previously, BHLHE40 expression was higher in lower-grade samples compared with higher-grade ones (Fig. 8*E*) (9, 42). BHLHE40 expression was higher in samples at stages I and II compared with those at stages III and IV (Fig. 8*F*) (9, 42). The expression levels of BHLHE40 and p-AMPKα were proportional (Figs. S7*A* and 8, *A*–*D*, and *G*). In contrast, the expression levels of BHLHE40 and PPM1F showed a reverse correlation (Figs. S7*B* and 8, *A–D*, and *H*). PPM1A expression levels were consistently high in all the samples and showed no correlation with any of the other three molecules (Fig. 8, *A*–*D*).

### Protein expression levels of BHLHE40 were correlated with the prognosis of patients with EC

The expression levels of BHLHE40, p-AMPKα, and PPM1F were analyzed for their correlation with the prognosis of EC patients. The Kaplan–Meier plotter analysis (https://kmplot.com/analysis/) from RNA-seq data showed that the group with higher expression of BHLHE40 had remarkably better progression-free survival than that with lower expression (Fig. 8*I*). The correlation between clinical and gene expression data from cBioPortal (https://www.cbioportal.org) was analyzed for PPM1F. Kaplan–Meier analysis showed that the group with lower expression of PPM1F had remarkably better progression-free survival (Fig. 8*J*). The clinical and IHC data from our institute was used for Kaplan–Meier analysis. The group with the higher total score of BHLHE40 had better progression-free and overall survival (Fig. 8*K*). This was also the case with p-AMPKα Thr172 (Fig. 8*L*). On the contrary, the group with the absence of PPM1F had better progression-free and overall survival (Fig. 8*M*).

## Discussion

BHLHE40 has been reported to have tumor suppressive functions (6, 9, 10, 42, 43, 44) and has been shown to affect energy metabolism by regulating PGC-1α, SREBP-1c, PKLR, PCK2, FASN, and PPARγ (11, 12, 13, 14, 15, 16). In this study, we elucidated a novel energy regulatory pathway of the BHLHE40‒PPM1F‒AMPKα axis in EC cells.

Regulation of AMPKα phosphorylation by BHLHE40 was previously reported by Sato *et al*. (34). They reported that BHLHE40 suppressed transcription of LKB1 and negatively regulated AMPKα phosphorylation at Thr172. However, our data indicated that BHLHE40 positively regulated the phosphorylation of AMPKα (Figs. 1, *B*–*E* and 3, *I–L*). This discrepancy may be caused by the cellular context. However, not only the *in vitro* data but also our data from clinical samples clearly suggested that the expression levels of BHLHE40 and phosphorylated AMPKα at Thr172 were proportional (Fig. 8, *A*–*D*, and *G*). Furthermore, our data showed that BHLHE40 did not have any effects on the expression and phosphorylation of LKB1 (Fig. 1, *B*–*E*).

GSEA showed that BHLHE40 signaling is involved in the AMPK, AKT, and glycolysis/glyconeogenesis pathways (Fig. 2, *A*–*D*). Previous studies also reported that BHLHE40 as well as AMPK is involved in AKT signaling (45, 46, 47, 48). Our proteomic analysis showed that knockdown of *BHLHE40* and the resulting suppression of AMPK activity enhanced the expression of enzymes involved in glycolysis and the pentose phosphate pathway (Fig. 2, *E* and *F*). These results were consistent with previous reports (18, 49).

Our flux analysis showed that BHLHE40 suppressed the ECAR and enhanced the OCR in EC cells (Fig. 1, *F–M*). Consistent with our results, Li *et al*. also reported that knockout of *Bhlhe40* in mouse tissue-resident memory CD8^+^ T (Trm) cells showed reduced OCR, TCA metabolites, and electron transport chain complex genes (17). They also found that the ECAR was enhanced in *Bhlhe40* knockout Trm cells (17). BHLHE40 not only promotes Trm cell commitment but also functions to sustain mitochondrial metabolism for survival. In tumor immunity, BHLHE40 also promotes the commitment of tumor-infiltrating lymphocytes (TILs), which resemble Trm cells (17). Because BHLHE40 is necessary for TILs to play a critical role in the immune system to suppress tumor growth, BHLHE40 acts as a tumor suppressor not only by functioning in tumor cells themselves but also by activating anti-tumor immune functions in the host. Therefore, a strategy to activate BHLHE40 signaling would be an effective way to control tumor development.

Enhanced ECARs and suppressed OCRs were also reported in murine T cell acute lymphoblastic leukemia cells or murine muscle stem cells in which the gene encoding AMPKα1 was knocked out (21, 22). Similarly, murine embryonic fibroblasts in which the genes encoding AMPKα1 or AMPKα2 were knocked out also showed enhanced ECARs and suppressed fatty acid oxidation (23). The findings above suggest that BHLHE40 regulates energy metabolism through AMPK signaling.

Our data indicated that phosphorylation of AMPKα induced by BHLHE40 enhanced PDH activity and suppressed LDH activity by regulating the phosphorylation status of PDHA1 at Ser293 and LDHA at Tyr10 (Fig. 3). It has been reported that inhibition or knockdown of *AMPKα* enhanced phosphorylation of PDHA1 at Ser293, suppressed PDH activity, suppressed production of TCA cycle metabolites, and suppressed electron transport chain (20, 36). These findings were consistent with our results (Fig. 4). Knockout of *AMPKα1/2* or knockdown of *AMPKα1* also enhanced the expression of PDK1, which is a well-known kinase that phosphorylates PDHA1 at Ser293. However, knockout or knockdown of *AMPKα* enhanced LDHA expression, LDH activity, ECAR, and lactate production (21, 24). Consistent with these findings, our data indicated that knockdown of *AMPKα* enhanced LDH activity and ECAR (Fig. 4). However, inconsistent with data from Faubert *et al*. studying, murine fibroblasts and lymphoma cells, our data showed knockdown of *AMPKα* did not alter LDHA expression (24) (Fig. 4, *A* and *B*). Knockout of *AMPKα1* in skeletal muscle stem cells enhanced LDH activity but did not alter LDHA expression (21). These data might suggest that altered expression and activity of LDHA depend on the cellular context. Our data also showed that the knockdown of *AMPKα* enhanced the phosphorylation of LDHA at Tyr10 (Fig. 4, *A* and *B*). Activation of LDHA was closely related to phosphorylation of LDHA at Tyr10 by SRC or HER2, promoting cell invasion and tumor cell metastasis, and was correlated with poorer prognosis (50, 51). The activation of SRC, HER2, and AMPKα phosphorylation at Thr172 was mutually exclusive (26, 52, 53). Moreover, active AMPK silenced the activity of HER2 and EGFR (54). Our data suggested that phosphorylation of LDHA at Tyr10 was regulated by AMPK activity controlled by BHLHE40. Furthermore, the expression of LDHA was altered by BHLHE40 but not by AMPK (Fig. 4, *A* and *B*). These results suggested that BHLHE40 suppressed the phosphorylation and activity of LDHA dependent on AMPK but that BHLHE40 suppressed the expression of LDHA independent of AMPK. This series of evidence is consistent with our results suggesting that AMPKα induced by BHLHE40 modulated PDH and LDH activity by regulating the phosphorylation status of PDHA1and LDHA (Fig. 3). Another group also recently reported that BHLHE40 suppressed LDH activity (55).

Our gel shift assay showed a clear E-box-BHLHE40 complex for promoter sequences of both *PPM1A* and *PPM1F* (Figs. 6, *A* and *B* and S4, *A* and *B*). However, ChIP assay revealed that with regards to *PPM1F*, the promoter regions containing E-box2 (−6602 to −6544 bp) and E-box4 (−4166 to −4089 bp) were associated with BHLHE40 and HDAC1 (Fig. 6*E*). In contrast, the *PPM1A* promoter region containing E-box1 (−1006 to −870 bp) showed no specific binding to BHLHE40 and HDAC1 (Fig. S4*D*). This discrepancy between the gel shift assay and ChIP assay may be caused by differences between reconstituted (gel shift assay) and native (ChIP assay) assay conditions. These results suggested that the effect of BHLHE40 on PPM1A expression may be indirect and mediated by another molecule located downstream of the BHLHE40 pathway.

In conclusion, we clarified the impact of BHLHE40 expression in the energy metabolism of EC cells. Our results suggested that BHLHE40 enhanced AMPK activity by suppressing an AMPK-specific phosphatase, PPM1F. BHLHE40 modulated the functions of AMPK in glycolysis and OXPHOS to shift energy dependency. Furthermore, BHLHE40 suppressed lactate production by dephosphorylating LDHA at Tyr10 and suppressing LDH activity. BHLHE40 also enhanced oxygen consumption by dephosphorylating PDHA1 at Ser293 and enhancing PDH activity. Because AMPK acts as a central regulator of energy metabolism in cancer cells, targeting the BHLHE40‒PPM1F‒AMPK axis may represent a strategy to control cancer development. PPM1F specific inhibitors, 1-amino-8-naphthol-2,4-disulfonic acid or 1-amino-8-naphthol-4-sulfonic acid may be therapeutic candidates (56).

## Experimental procedures

### Cell lines

293T, HHUA, HEC-1, Ishikawa, HEC-6, and AN3 CA cells were cultured in DMEM supplemented with 10% fetal bovine serum, penicillin, and streptomycin. KLE cells were cultured in DMEM:F-12 supplemented with 10% fetal bovine serum, penicillin, and streptomycin. Cells were cultured at 37 °C and in 5% CO_2_ atmosphere. HEC-1 and HEC-6 cells were purchased from the Japanese Collection of Research Bioresources. HHUA cells were purchased from the RIKEN BioResource Center. 293T cells were from Invitrogen. Ishikawa cells were purchased from Sigma-Aldrich. AN3 CA and KLE cells were purchased from the American Type Culture Collection. 293T, Ishikawa, AN3CA, and KLE cells were used within seven passages. The authentication of HHUA, HEC-1, and HEC-6 cells was confirmed using DNA profiling provided by the Japanese Collection of Research Bioresources Cell Bank. We confirmed that all the cell lines were free from contamination of *mycoplasma*.

### Patient recruitment and tissue samples

Thirty-nine EC patients who had surgery at Kyushu University Hospital between 2010 and 2015 were involved in this study. The 39 EC primary tissue samples from 13 cases at stage IA, 8 at stage IB, 4 at stage II, 2 at stage IIIA, 1 at IIIB, 3 at stage IIIC1, 6 at IIIC2, and 2 at stage IVB based on the surgical staging of International Federation of Gynecology and Obstetrics 2008 were examined by immunohistochemistry. On histological grading, 31 cases were endometrioid carcinoma including 17 at grade 1, 10 at grade 2, and 4 at grade 3, and 8 cases were serous carcinoma. This study was conducted according to the principle of the Declaration of Helsinki and was authorized by the Ethical Committee of Kyushu University (approval No. 1–2). All study participants provided informed written consent prior to study enrollment.

### Real-time reverse transcription (RT)-qPCR assay

Total RNA was extracted from cultured cells using an RNeasy Mini Kit (QIAGEN). Complementary DNA was synthesized using a ReverTra Ace kit (Toyobo). Real-time PCR was performed using Sso Advanced Universal SYBR Green Supermix and a CFX Connect Real-Time PCR Detection System. The information on used primer sets is shown in Table S1. The relative expression levels of target genes were determined after standardization against those of *ACTB*. We designed all primers to locate across an intron.

### Immunohistochemistry

Immunohistochemistry was performed using the following antibodies: anti-BHLHE40 (HPA028921; Atlas Antibodies), anti-phospho-AMPKα (Thr172) (40H9; Cell Signaling Technology), anti-PPM1A (D18C10), or anti-PPM1F antibody (ab200394; Abcam). Briefly, a paraffin-embedded block was sliced into 5-μm-thick sections. The sections were deparaffinized and antigen was retrieved by Target Retrieval Solution (Agilent Dako; pH6 for BHLHE40 and pH9 for phospho-AMPKα, PPM1A, and PPM1F), then endogenous peroxidase was blocked with 0.3% hydrogen peroxide in methanol. The sections were incubated overnight with diluted primary antibody (1/200 in Antibody Diluent; Agilent Dako), then incubated with Envision+ Dual Link HRP (Agilent Dako) and visualized with 3, 3′ diaminobenzidine as a substrate. Hematoxylin was used for counterstaining. Specific staining was evaluated by the Allred scoring system (57).

### Immunoblotting

Cell lysates for immunoblotting were prepared with cell lysis buffer (20 mM HEPES, pH 7.9, 0.5 N NaCl, 1 mM EDTA, 25% glycerol, 1% Ninodet P-40, 0.5 mM dithiothreitol, and 0.1% sodium deoxycholate) containing protein inhibitor and phosphatase inhibitor (Nacalai Tesque). After separation by electrophoresis, the proteins were transferred to the PVDF membrane (Immobilon, Merck Millipore). Immunoblotting was performed using the following primary antibodies: anti-BHLHE40 (HPA028921) from Atlas Antibodies, anti-AMPKα (D5A2), anti-phospho-AMPKα Thr172 (40H9), anti-AMPKβ (57C12), anti-phospho-AMPKβ1 Ser182 (4186), anti-ACC (C83B10), anti-phospho-ACC Ser79 (D7D11), anti-PPM1A (D18C10), anti-PDHA1 (C54G1), anti-phospho-PDHA1 Ser293 (31,866), anti-LDHA (C4B5), anti-phospho-LDHA Tyr10 (8167), anti-LKB1 (D60C5), anti-phospho-LKB1 Ser428 (C67A3), and anti-β-actin (13E5) (Cell Signaling Technology). Anti-PPM1B (ab70804), anti-PPM1E (ab137122), and anti-PPM1F (ab200394) antibodies were from Abcam. Anti-GAPDH (FL-335) antibody was from Santa Cruz Biotechnology. Anti-FLAG (M5) antibody was from Sigma–Aldrich (9). The intensity of blotting was semi-quantified using Image J software (https://imagej.net/ij/).

### Plasmid transfection, lentivirus vector infection, and reporter assay

HA- or FLAG-tagged human BHLHE40 open reading frames were amplified by PCR using cDNA from HHUA cells and inserted into pCDNA3 and pENTR4 vectors. HA- or FLAG-tagged human BHLHE40 was re-ligated into a pLX302 vector. Short hairpin RNA (shRNA) sequences for *BHLHE40* (shBHLHE40–1 and shBHLHE40–2) were from Sigma–Aldrich (Mission shRNA validated sequences) and were ligated into a pLKO.1-puro vector (Addgene, Cambridge, MA, USA). The pLX302 and pLKO.1-pruro vectors generated were used with envelope and packaging vectors (Addgene) to produce lentivirus vectors in 293T cells (Invitrogen). The lentivirus vectors generated were transduced into EC cells to express BHLHE40 or knock down *BHLHE40*. The transduced cells were selected by puromycin. shRNA target sites and sequences are shown in Table S2. Both shRNAs for *BHLHE40* showed similar efficiencies (Fig. 1, *B* and *C*) and shBHLHE40 to 2 for *BHLHE40* was used in most cases.

pCDNA3.1 vectors to express FLAG-tagged human PPM1A (OHu29195), PPM1B (OHu16270), PPM1E (OHu08016), and PPM1F (OHu08331) were purchased from GenScript (Piscataway). PPM1A-R174G and PPM1F-R326A-I328A mutants were constructed by PCR-based mutagenesis. The primer information to generate the mutants is shown in Table S3.

DNA regions upstream of *PPM1A* (spanning −1098 bp to +820 bp from the transcription start site) and *PPM1F* (spanning −7558 bp to −6090 bp and −4288 bp to −3179 bp from the transcription start site) were generated by PCR and inserted into a pGL4.22-basic luciferase vector (Promega). The primer information for mutagenesis is shown in Table S3. In reporter assays, cells (1 × 10^5^) were transfected with 100 ng of each luciferase reporter, 100 ng expression vector or 10 pmol siBHLHE40 (sc-106769, Santa Cruz Biotechnology), and 5 ng pRL-tk vector (Promega) using Lipofectamine 3000 reagent (Invitrogen). Cell lysates were collected 24 h after transfection, and assayed using a Dual-Luciferase Reporter Assay System kit (Promega). Activity values of firefly luciferase were standardized against those of *Renilla* luciferase. The DNA sequence of each construct was confirmed by a sequence reaction using an ABI PRISM BigDye Terminator v3.1 Cycle Sequencing Kit (Applied Biosystems).

### siRNA transfection

Double-stranded small interfering RNAs (siRNAs) against BHLHE40 (sc-106769), AMPKα1/2 (sc-45312), PPM1A (sc-45214), PPM1B (sc-61387), PPM1E (sc-62842), PPM1F (sc-62844), and control siRNA (sc-37007) were purchased with from Santa Cruz Biotechnology. siRNA was transfected into cells at a final concentration of 50 nM using Lipofectamine 3000 reagent (Invitrogen).

### Microarray analysis

Whole-genome expression analysis was conducted using SurePrint G3 Human Gene Expression Microarrays 8 × 60 K version 3 (Agilent Technologies). Briefly, total RNA from HHUA cells cultured in DMEM containing 1.0 g/L glucose with 10% FBS was extracted using an RNeasy Mini Kit. In total, 50 ng total RNA was labeled with the Agilent Low-Input QuickAmp Labeling Kit. Relative target intensity was quantified using Agilent Feature Extraction software (Agilent Technologies). The data were registered at the National Center for Biotechnology Information (https://www.ncbi.nlm.nih.gov/geo/; accession number GSE241941). Gene Set Enrichment Analysis (https://www.gsea-msigdb.org/gsea/index.jsp) of the microarray data was performed to evaluate enrichment of downregulated genes from GSE97735 and GSE147470.

### Large-scale absolute quantitative proteomics analysis

Absolute quantitative protein expression analysis of 342 main metabolic enzymes was performed by *in vitro* proteome-assisted multiple reaction monitoring for protein absolute quantification (iMPAQT) assay, as previously described (35). Briefly, 2 × 10^6^ HHUA cells cultured in DMEM with 10% FBS with 1 mM sodium pyruvate without glucose for 24 h were lysed in lysis buffer (100 mM Tris-HCl, pH8.8, 2% SDS, and 7 M urea) and sonicated using a sonicator (Bioruptor, Diagenode). The protein concentration of the lysates was determined by BCA assay (Thermo Fisher Scientific). Reactions including 200 μg protein were treated with 10 mM Tris (2-carboxyethyl) phosphine HCl for 45 min at 37°C to break S–S bonds and then alkylated with 20 mM 2-iodoacetoamide for 30 min at room temperature. After acetone precipitation, the pellets were suspended in 100 μl digestion buffer (50 mM triethylammonium bicarbonate and 7M guanidine hydroxide) and digested with lysyl-endopeptidase for 3 h at 37 °C and then with trypsin overnight at 37 °C. The cell digests were freeze-dried and labeled with mTRAQ Δ0 reagent (SCIEX). Each sample was spiked with synthetic peptides for the internal standard, reductively alkylated, and labeled with mTRAQ Δ4 reagent (SCIEX). The labeled peptide mixtures were fractionated by revise-phase liquid chromatography. The high-performance LC system was coupled with a TripleTOF5600 hybrid mass spectrometer (SCIEX). Multiple-Reaction Monitoring (MRM) analysis was performed using a QTRAP6500 instrument operated in positive-ion mode. Pretreatment of the samples and assay using mass spectrometry and MRM analysis were performed by Kyushu Pro Search LLP. A heat map, hierarchical clustering, and volcano plotting were generated using the MetaboAnalystR package (https://www.metaboanalyst.ca).

### Extracellular flux analysis

OCRs and ECARs of the cells were measured with an XFp Extracellular Flux Analyzer (Agilent Technologies). In brief, cells were plated at a density of 2 × 10^4^/well in 80 μl Seahorse XF DMEM medium. OCR was measured in the presence of oligomycin (1.0 μM), FCCP (1.0 μM for HHUA; 0.25 μM for KLE; 2.0 μM for HEC-1 and Ishikawa cells), and rotenone/antimycin A (0.5 μM). ECAR was measured in the presence of glucose (10 mM), oligomycin (1.0 μM), and 2-deoxyglucose (50 mM). Data were corrected by total protein weight.

### PDH and LDH activity assay, ADP/ATP ratio assay, and lactate detection assay

EC cells cultured for 24 h in DMEM or DMEM:F12 with 10% FBS with 1 mM sodium pyruvate without glucose were applied to the Pyruvate Dehydrogenase Activity Colorimetric Assay (K609-100; BioVision), Lactate Dehydrogenase Activity Colorimetric Assay (K726-500, BioVision), and ADP/ATP Ratio Assay (MAK135, Sigma–Aldrich) according to the manufacturers’ instructions. EC cell lysates and conditioned medium after culturing for 12 h in DMEM or DMEM:F12 with 1 mM sodium pyruvate without glucose and with dialyzed FBS (A33820-01; Thermo Fisher Scientific) were applied to the Lactate-Glo Assay (J5021; Promega) according to the manufacturer’s instruction. PDH and LDH activity values were corrected by cell numbers and lactate detection values were corrected by values from the CellTiter 96 AQuous One Solution Proliferation Assay (Promega).

### Gel shift assay

Gel shift assays were conducted using labeled DNA probes and nuclear extracts from 293T cells expressing FLAG-BHLHE40. Synthesized oligonucleotides were annealed and labeled at the 3′ end with digoxigenin-11-ddUTP using a terminal transferase (Roche Diagnostics). For isolation of nuclear extracts, 293T cells were lysed in cell lysis buffer (10 mM HEPES-KOH, pH 7.9, 10 mM KCl, 0.1 mM EDTA, 0.1 mM EGTA, 1 mM DTT, 0.625% Nonidet P-40, and 1 mM PMSF). After centrifugation, nuclear extracts were lysed in nuclear lysis buffer (20 mM HEPES-KOH, pH 7.9, 400 mM NaCl, 1 mM EDTA, 1 mM EGTA, 1 mM DTT, and 1 mM PMSF). Five micrograms of nuclear extract was incubated with 0.4 ng digoxigenin-labeled oligonucleotide probe in binding buffer (10 mM HEPES-KOH, pH 7.9, 50 mM KCl, 2.5 mM MgCl_2_, 10% glycerol, and 1 mM DTT) for 20 min at room temperature. After binding, the reactions were electrophoresed on 4% native polyacrylamide gels and transferred to a Hybond N+ hybridization membrane (Merck Millipore). The membrane was cross-linked at 120 mJ, and reacted with anti-digoxigenin-AP antibody (Roche Diagnostics). Signals of the digoxigenin-labeled oligonucleotide the probe–protein complexes were detected using CSPD chemiluminescent substrate (Roche Diagnostics). The specificity of the probe–protein complexes was confirmed by undertaking the binding reaction in the presence of excess amounts of unlabeled wild-type or mutant oligonucleotide competitors. The presence of FLAG-BHLHE40 in the binding complex was confirmed through the formation of supershift bands with anti-FLAG antibody (Sigma–Aldrich). An anti-SRF antibody (Santa Cruz Biotechnology) was used as a negative control. The sequence information of the probes used is shown in Table S4.

### ChIP assay

ChIP assays were conducted as follows. 293T cells expressing FLAG-BHLHE40 were fixed with 0.4% formaldehyde and neutralized with 125 mM glycine. The cells were harvested by scraping and then lysed with nuclear lysis buffer (50 mM Tris-HCl, pH 8.0, 10 mM EDTA, and 1% SDS). The nuclear lysates were sonicated using a Bioruptor II (Diagenode) to produce chromatin fragments at a size of approximately 500 bp. After DNA–protein complexes diluted in IP dilution buffer (20 mM Tris-HC, pH 8.0, 150 mM NaCl, 2 mM EDTA, 1% Triton X-100, and 0.01% SDS) were precleared using mouse or rabbit IgG with protein G Plus-Agarose (Santa Cruz Biotechnology), they were incubated overnight with anti-HA (HA-7; Sigma–Aldrich), anti-HA (ab9110; Abcam), anti-acetylated histone H3 (Merck Millipore), anti-HDAC1 (ab7028; Abcam), or anti-PCAF (ab12188; Abcam) antibodies with protein G Plus-Agarose (Santa Cruz Biotechnology). Rabbit IgG was used for non-specific binding. Immunoprecipitated chromatin with agarose was washed with IP wash buffer 1 (20 mM Tris-HCl, pH 8.0, 50 mM NaCl, 2 mM EDTA, 1% Triton X-100, and 0.1% SDS) and IP wash buffer 2 (10 mM Tris-HCl, pH 8.0, 1% w/v LiCl, 1 mM EDTA, 1% Nonidet P-40, and 1% w/v sodium deoxycholate). Eluted DNA–protein complexes in elution buffer (0.84% w/v NaHCO_3,_ 1% SDS) were reverse cross-linked with 300 mM NaCl at 65°C for 4 h. After treatment with RNase A and proteinase K, DNA fragments were extracted with phenol–chloroform and ethanol. DNA fragments from immunoprecipitated samples were used for qPCR to semi-quantify the E-box and negative control regions of the *PPM1A* and *PPM1F* promoters with the primers shown in Table S1.

### *In vitro* PPM1A and PPM1F phosphatase assay

Cell lysates of 293T transfected with FLAG-tagged constructs of PPM1A-wild type, PPM1A-R174G, PPM1F, and PPM1F-R326A-I328A were precleared with mouse IgG and A/G-agarose suspensions (Santa Cruz). Then the lysates were incubated with anti-FLAG antibody (M5) and protein A/G-agarose suspensions (Santa Cruz). Immune complexes bound to protein A/G-agarose were precipitated and incubated at 30°C for 30 min with recombinant active AMPK (α1β1γ2) (PV6238; Thermo Fisher Scientific) in 1× NEB buffer for PMP and 1 mM MnCl_2_ (P0753S; New England Biolabs). Lambda Protein Phosphatase was used as a positive control (P0753S). The reaction mixtures were separated on 9% SDS-polyacrylamide gels and transferred to PVDF membranes. Phosphorylated AMPKα1 was immunodetected with anti-phospho-AMPKα Thr172 (40H9; Cell Signaling Technology).

### Statistical analysis

Data are presented as the mean ± standard deviation (SD). Comparison between case-control data was analyzed using two-sided Student’s *t* test or the Mann–Whitney *U* test. Welch’s test was applied when heteroscedasticity of the data was suspected. The correlation of two-group data was evaluated using Pearson’s product-moment correlation coefficient. Statistical analysis of the relationships was conducted using the *F*-test. A *p*-value of <0.05 was considered significant. Correlations between the total staining score of our immunohistochemical (IHC) analysis and the prognosis of patients (n = 39) were visualized by a Kaplan–Meier curve and evaluated by the log-rank test. Correlations between *BHLHE40* mRNA levels and recurrence-free survival of EC cases (n = 543 from Gene Expression Omnibus, European Genome-Phenome Archive, and The Cancer Genome Atlas (TCGA) databases) were analyzed by KM potter (http://kmplotter.com/analysis/). Correlations between *PPM1F* mRNA and progression-free survival from TCGA (n = 505) was visualized by a Kaplan–Meier curve and evaluated by the log-rank test.

## Data availability

All data in this study are provided within this paper, supporting information, and from the corresponding author on request.

## Supporting information

This article contains supporting information (58).

## Conflict of interest

The authors declare that they have no conflicts of interest with the contents of this article.
