# Supplementary material for: The BHLHE40‒PPM1F‒AMPK pathway regulates energy metabolism and is associated with the aggressiveness of endometrial cancer
Source: J Biol Chem. 2024 Jan 30;300(3):105695. doi: 10.1016/j.jbc.2024.105695 (PMC10904277; doi:10.1016/j.jbc.2024.105695)
Supplement: Supplemental table [file mmc1.docx]

**Table S1: Primers used for RT-qPCR and ChIP-qPCR analyses**

| **Symbol** | **Accession No.** | **Forward primer** | **Reverse primer** | **Amplicon (bp)** |
| --- | --- | --- | --- | --- |
| PPM1A | NM_021003 | 5’-GCACCCAAAGTATCGCCAGA-3’ | 5’-GCCTTCCCCCTGCTTCTTTA-3’ | 99 |
| PPM1E | NM_014906 | 5’-CAAAGGAAACGGATGGCACA-3’ | 5’-CCAGCTGCAGCAAATCTCGT-3’ | 98 |
| PPM1F | NM_014634 | 5’-CTCAGGAAAGCCAAGCGAGA-3’ | 5’-CTTCACCACCTGTCCCTGCT-3’ | 135 |
| ACTB | NM_001101 | 5’-TTGCCGACAGGATGCAGAAG-3’ | 5’-CAGCGAGGCCAGGATGGAGC-3’ | 122 |
| pPPM1A: −1006–−870 |  | 5’-GCTGGCACGTGCAGTTTCTA-3’ | 5’-GGACCTCCAACGACCTCCAA-3’ | 137 |
| pPPM1A: +2261–+2351 |  | 5’-GTCATGGCTGTGCTCCAAAA-3’ | 5’-TGCCCAACTGGCTTTACTGA-3’ | 91 |
| pPPM1F: −6602–−6544 |  | 5’-ACCTCCTGGCTGGGTTGAAA-3’ | 5’-ACGTGTTCCTGGCCATGTGT-3’ | 59 |
| pPPM1F: −4166–−4089 |  | 5’-CTTTGCTGGTGCAGCGATGT-3’ | 5’-TGGGTTTCGGAGAGGGAGAG-3’ | 78 |
| pPPM1F: −375–−252 |  | 5’-CCTGGGACTGTGCCTCCTTC-3’ | 5’-TAGGTCACAGCGCCCAGAAA-3’ | 124 |

**Table S2: Sequences of shRNA constructs**

| **shRNA name** | Target | **Target site** | shRNA sequence (cloned into AgeI and EcoRI) |
| --- | --- | --- | --- |
| Control shRNA (shCtrl) | None | CCTAAGGTTAAGTCGCCCTCG | 5'-accggtCCTAAGGTTAAGTCGCCCTCGCTCGAG CGAGGGCGACTTAACCTTAGGTTTTTTTgaattc-3' |
| shBHLHE40-1 (TRCN0000013249) | BHLHE40 Exon 4 | GCACTAACAAACCTAATTGAT | 5'-accggtGCACTAACAAACCTAATTGATCTCGAG ATCAATTAGGTTTGTTAGTGCTTTTTTgaattc-3' |
| shBHLHE40-2 (TRCN0000232187) | BHLHE40 Exon 4 | CATGTGAAAGCACTAACAAAC | 5'-accggtGCATGTGAAAGCACTAACAAACCTCGAG GTTTGTTAGTGCTTTCACATGCTTTTTTgaatttc-3' |

Cloning sites are shown in lowercase letters.

**Table S3: Sequences of forward primers used to generate mutant reporter constructs**

| **Reporter** | **Sense oligonucleotide** |
| --- | --- |
| PPM1A-R174G | +514 AAAGAAGGAATTCAGAATGCAGGTGGCTCTGTAATGA +550 |
| PPM1F-R326A | +970 GTCTCCGCCGCCATCGGGGATGTCTTCCAGAAGCCCT +1006 |
| PPM1F-R326A-I328A | +976 GCCGCCGCCGGGGATGTCTTCCAGAAGCCCTACGTGTCT +1014 |
| pPPM1A-wt E-box1 | −1004 TGGCACGTGCAGTTTCTATTAACTTTAGCTCATCTCTACCT −964 |
| pPPM1A-mut E-box1 | −1004 TGGAAAATGCAGTTTCTATTAACTTTAGCTCATCTCTACCT −969 |
| pPPM1A-wt E-box2 | −859 AACCACGTGATTATCTAACATGTGCAAATAAATACAATGTGGA −817 |
| pPPM1A-mut E-box2 | −859 AACAAAATGATTATCTAACATGTGCAAATAAATACAATGTGGA −817 |
| pPPM1F-wt E-box2 | −6551 GAACACGTGCCTCTGACGCACAGGGCT −6525 |
| pPPM1F-mut E-box2 | −6551 GAAAAAATGCCTCTGACGCACAGGGCT −6525 |
| pPPM1F-wt E-box4 | −4067 ATACACGTGCCCA GAGTGCTCCCTGCTA −4040 |
| pPPM1F-mut E-box4 | −4067 ATAAAAATGCCCAGAGTGCTCCCTGCTA −4040 |

Mutated nucleotides are underlined.

**Table S4: Sequences of oligonucleotide probes**

| **Probe** | **Sense oligonucleotide** |
| --- | --- |
| pPPM1A-wt E-box1 | −1011 TGGCCGCTGGCACGTGCAGTTTCTA −987 |
| PPM1A-mut E-box1 | −1011 TGGCCGCTGGAAAATGCAGTTTCTA −987 |
| pPPM1A-E-box2 | −867 TGGGACAGAACCACGTGATTATCTAA −842 |
| pPPM1F-E-box1 | −6670 ACCCAGGTCCCCACGTGGGACCAGCT −6645 |
| pPPM1F-wt E-box2 | −6558 TGGCCAGGAACACGTGCCTCTGACGCA −6532 |
| pPPM1F-mut E-box2 | −6558 TGGCCAGGAAAAAATGCCTCTGACGCA −6532 |
| pPPM1F- E-box3 | −6461 ACCCTTGACCACGTGTTGCGGAGCCT −6436 |
| pPPM1F-wt E-box4 | −4074 AGCCTGCATACACGTGCCCAGAGTGCT −4048 |
| pPPM1F-mut E-box4 | −4074 AGCCTGCATAAAAATGCCCAGAGTGCT −4048 |
| pBHLHE41-E-box (positive control) | +36 CGTTCCGCACGTGAGCTGGG +55 |

Altered nucleotides are underlined.
