# Supplementary material for: The BHLHE40‒PPM1F‒AMPK pathway regulates energy metabolism and is associated with the aggressiveness of endometrial cancer
Source: J Biol Chem. 2024 Jan 30;300(3):105695. doi: 10.1016/j.jbc.2024.105695 (PMC10904277; doi:10.1016/j.jbc.2024.105695)
Supplement: Supplemental Figure [file mmc2.docx]

**The BHLHE40**‒**PPM1F**‒**AMPK pathway regulates energy metabolism and is associated with the aggressiveness of endometrial cancer**

Kazuo Asanoma, Hiroshi Yagi, Ichiro Onoyama, Lin Cui, Emiko Hori, Minoru Kawakami, Shoji Maenohara, Kazuhisa Hachisuga, Hiroshi Tomonobe, Keisuke Kodama, Masafumi Yasunaga, Tatsuhiro Ohgami, Kaoru Okugawa, Hideaki Yahata, Hiroyuki Kitao, Kiyoko Kato

**Supporting Information Figures**

Fig. S1

Fig. S2

Fig. S3

Fig.S4

Fig. S5

Fig. S6

Fig. S7


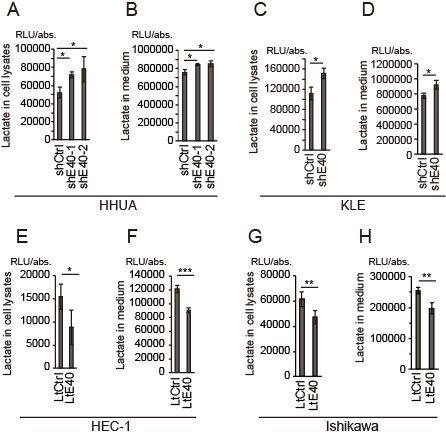


**Fig. S1.**

BHLHE40 suppressed lactate production in EC cells. (**A–H**) Lactate concentration in cell lysates (**A, C, E, G**) and conditioned medium (**B, D, F, H**) of HHUA (**A, B**), KLE (**C, D**), HEC-1 (**E, F**), and Ishikawa (**G, H**) cells after culturing for 12 hours. Data are from five technical replicates. The experiments were biologically replicated three times and representative data are shown. shCtrl, shControl; shE40, shBHLHE40; LtCtrl, LtControl; LtE40, LtBHLHE40; Unpaired two-sided Student’s *t*-test or the Mann–Whitney *U* test was used. *, *P*<0.05; **, *P*<0.01; ***, *P*<0.001.


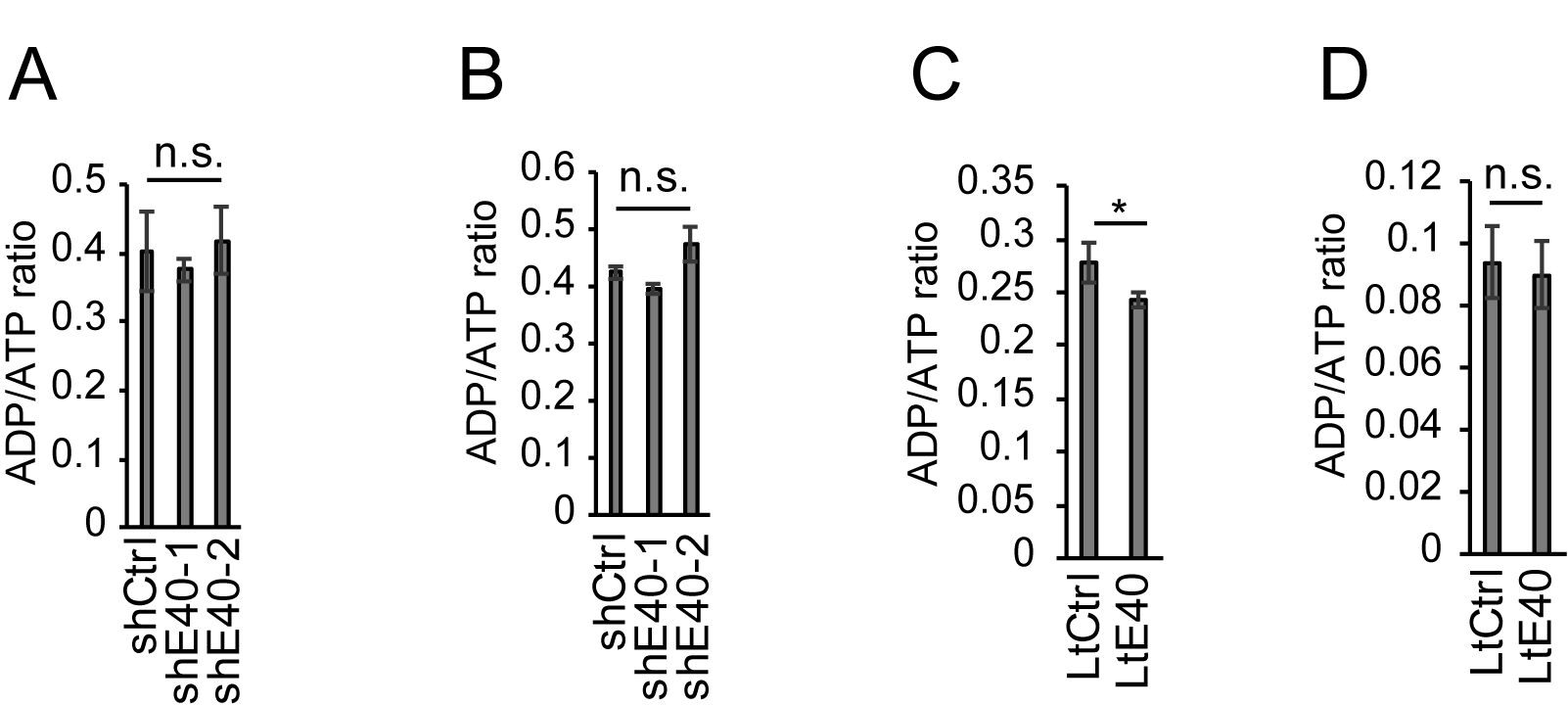
**Fig. S2.**

BHLHE40 did not affect the ADP/ATP ratio in EC cells. (**A–H**) ADT/ATP ratio in cell lysates (**A–D**) of HHUA (**A**), KLE (**B**), HEC-1 (**C**), and Ishikawa (**D**) cells after culturing for 12 hours. Data are from three technical replicates. The experiments were biologically replicated three times and representative data are shown. shCtrl, shControl; shE40, shBHLHE40; LtCtrl, LtControl; LtE40, LtBHLHE40; Unpaired two-sided Student’s *t*-test or the Mann–Whitney *U* test was used. n.s., not significant; *, *P*<0.05.


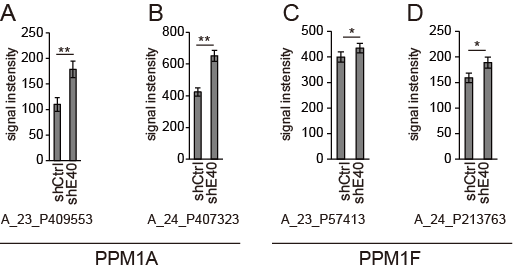


**Fig. S3.**

BHLHE40 enhanced mRNA expression of *PPM1A* and *PPM1F* on DNA microarray analysis. Results for *PPM1A* (**A, B**) and *PPM1F* (**C, D**) using the indicated probes. shCtrl, shControl; shE40, shBHLHE40; n.s., not significant; Unpaired two-sided Student’s *t*-test was used. *, *P*<0.05; **, *P*<0.01.


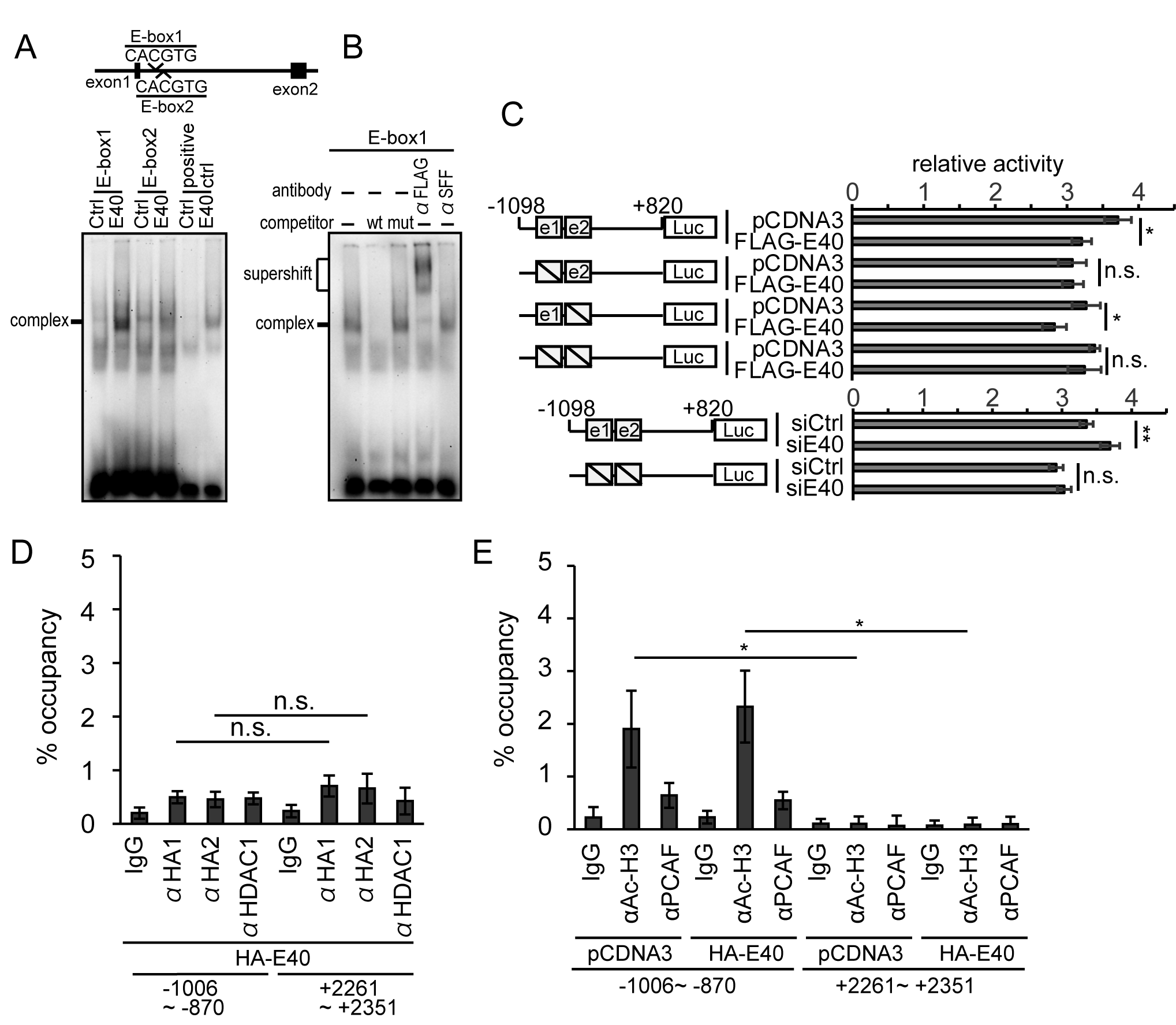


**Fig. S4.**

BHLHE40 transcriptionally suppressed PPM1A expression. (**A, top**) Schematic presentation of four E-boxes in the promoter of *PPM1A*. (**A, bottom**) Gel shift assay using nuclear extracts from 293T cells transfected with FLAG-BHLHE40 were incubated with labeled E-box1 and 2 probes (Table S4). A canonical E-box probe from the *BHLHE41* promoter was used as a positive control (1). (**B**) Anti-FLAG antibody was used to form supershifted bands. An anti-SRF antibody was used as a negative control. (**A, B**) Data are representative of two biological replicates. (**C, top**) Reporter analysis of the wild type and mutant *PPM1A* promoter in HEC-6 cells transfected with FLAG-BHLHE40 (Table S3). (**C, bottom**) Reporter analysis of the wild type and mutant *PPM1FA* promoter in HHUA cells transfected with siBHLHE40 at a concentration of 50 nM. See also Fig. S6. (**C**) Data are from four technical replicates. The experiments were biologically replicated three times and representative data are shown. (**D, E**) ChIP assay using 293T cells transfected with empty vector (pCDNA3) or HA-BHLHE40 (pCDNA3-HA-BHLHE40). Protein–DNA complexes immunoprecipitated with an anti-HA, anti-HDAC1, anti-acetylated-histone H3 (Ac-H3), or anti-PCAF antibody were used to amplify indicated promoter regions by PCR (Table S1). The −1006–−870 region contains E-Box1 and E-Box2. The +2261–+2351 region represents the negative control. The 10% input samples were used to calculate the occupancy ratio (%) from the values measured by real-time PCR. αHA1, anti-HA (HA-7, Sigma-Aldrich) antibody; αHA2, anti-HA (ab9110, Abcam) antibody. (**D, E**) Data are from three biological replicates. (**C–E**) Unpaired two-sided Student’s *t*-test or the Mann–Whitney *U* test was used. n.s., not significant; *, *P*<0.05.


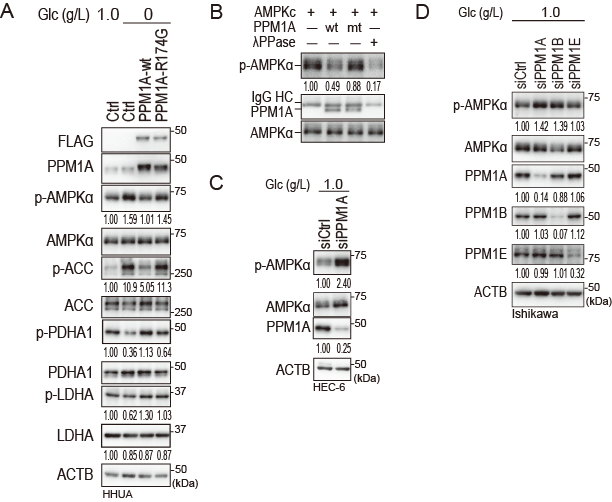
**Fig. S5.**

Phosphatase activity of PPM1s on phospho-AMPKα Ser172. (**A**) Immunoblotting of HHUA cells transfected with wild type and phosphatase inactive mutant (R174G) PPM1A. (**B)** In vitro phosphatase assay reconstituted with FLAG-PPM1A and activated AMPK complex. (**C**, **D**) Immunoblotting analysis of HEC-6 (**C**) and Ishikawa (**D**) cells transfected with the indicated siRNAs. Values under panels indicate relative expression levels of p-AMPKα/AMPKα, p-ACC/ACC, p-PDHA1/PDHA1, p-LDHA/LDHA, LDHA/ACTB, PPM1A/ACTB, PPM1B/ACTB and PPM1E/ACTB. Data are representative of at least two technical replicates from three biological replicates.


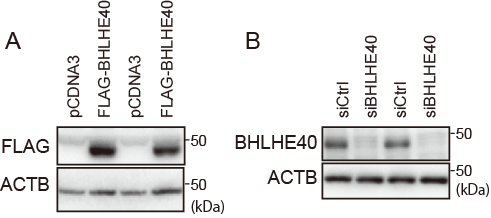


**Fig. S6.**

Examples of the immunoblotting analysis showing that the samples used in the reporter assays in Figure 5 and Supplementary Figure 3 expressed similar amounts of BHLHE40. (**A**) Immunoblotting analysis of HEC-6 cells used in the reporter assays showing similar amount of FLAG-BHLHE40. (**B**) Immunoblotting analysis of HHUA cells used in reporter assays showing similarly reduced expression of BHLHE40.


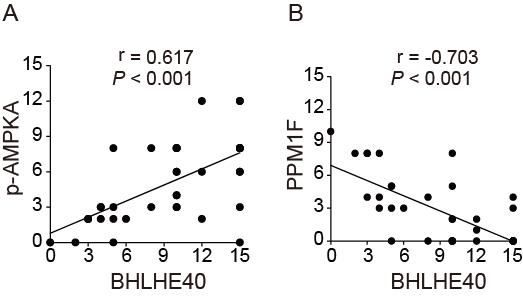


**Fig. S7.**

Pearson’s product-moment correlation coefficients were recalculated using staining scores produced by multiplying the proportion score by the intensity score (related to Fig. 8G, H). (**A**) Staining scores of BHLHE40 and phospho-AMPKα were analyzed using Pearson’s product-moment correlation coefficient. (**B**) Staining scores of BHLHE40 and PPM1F were analyzed using Pearson’s product-moment correlation coefficient. r-values show correlation coefficients.

1. Li, Y., Xie, M., Song, X., Gragen, S., Sachdeva, K., Wan, Y. *et al.* (2003) DEC1 negatively regulates the expression of DEC2 through binding to the E-box in the proximal promoter *J Biol Chem*. **278**, 16899-16907 10.1074/jbc.M300596200
